# A new Graph Gaussian embedding method for analyzing the effects of cognitive training

**DOI:** 10.1371/journal.pcbi.1008186

**Published:** 2020-09-17

**Authors:** Mengjia Xu, Zhijiang Wang, Haifeng Zhang, Dimitrios Pantazis, Huali Wang, Quanzheng Li

**Affiliations:** 1 Division of Applied Mathematics, Brown University, Providence, Rhode Island, United States of America; 2 McGovern Institute for Brain Research, Massachusetts Institute of Technology, Cambridge, Massachusetts, United States of America; 3 Peking University Institute of Mental Health (Sixth Hospital), Beijing, China; 4 National Clinical Research Center for Mental Disorders & Key Laboratory of Mental Health, Ministry of Health, Peking University, Beijing, China; 5 Beijing Municipal Key Laboratory for Translational Research on Diagnosis and Treatment of Dementia, Beijing, China; 6 Massachusetts General Hospital and Harvard Medical School, Boston, Massachusetts, United States of America; Emory University, UNITED STATES

## Abstract

Identifying heterogeneous cognitive impairment markers at an early stage is vital for Alzheimer’s disease diagnosis. However, due to complex and uncertain brain connectivity features in the cognitive domains, it remains challenging to quantify functional brain connectomic changes during non-pharmacological interventions for amnestic mild cognitive impairment (aMCI) patients. We present a *quantitative* method for functional brain network analysis of fMRI data based on the multi-graph unsupervised Gaussian embedding method (MG2G). This neural network-based model can effectively learn low-dimensional Gaussian distributions from the original high-dimensional sparse functional brain networks, quantify uncertainties in link prediction, and discover the *intrinsic* dimensionality of brain networks. Using the Wasserstein distance to measure probabilistic changes, we discovered that brain regions in the default mode network and somatosensory/somatomotor hand, fronto-parietal task control, memory retrieval, and visual and dorsal attention systems had relatively large variations during non-pharmacological training, which might provide distinct biomarkers for fine-grained monitoring of aMCI cognitive alteration. An important finding of our study is the ability of the new method to capture subtle changes for individual patients before and after short-term intervention. More broadly, the MG2G method can be used in studying multiple brain disorders and injuries, e.g., in Parkinson’s disease or traumatic brain injury (TBI), and hence it will be useful to the wider neuroscience community.

## Author summary

There is still no cure and no effective drug treatment for Alzheimer’s disease (AD). Hence, *non-pharmacological* cognitive intervention for patients at early stages of AD has received a lot of attention due to its non-invasive manner, safety, and scalability. Multi-domain interventions targeting memory and non-memory domains simultaneously are urgently needed for an optimal intervention effect on amnestic mild cognitive impairment (aMCI) patients. However, most of the previous multi-domain cognitive intervention studies evaluated the intervention outcomes based solely on neuropsychological assessment or simple characterization of brain anatomical structural changes. This work is the first of its kind to develop a patient-specific *quantitative analysis* for the underlying *functional* brain regional activity changes during the multi-domain cognitive intervention process. Specifically, we used fMRI data from 12 patients, who were trained for three months, and we developed the multi-graph unsupervised Gaussian embedding method (MG2G) to analyze these data. We obtained probabilistic changes across all the brain regions, and we found that brain regions in the default mode network and somatosensory/somatomotor hand, fronto-parietal task control, memory retrieval, and visual and dorsal attention systems had relatively large variations during the non-pharmacological training. These fundamental insights could provide effective new biomarkers for monitoring of aMCI cognitive alteration.

This is a *PLOS Computational Biology* Methods paper.

## Introduction

Alzheimer’s disease (AD) is a neurodegenerative brain disorder and the most common form of dementia. However, there is still no cure and no effective drug treatment for AD [1, 2]. Hence, non-pharmacological cognitive intervention for patients at early stages of AD has received a lot of attention due to its non-invasive manner, safety and scalability. Recent studies show that non-pharmacological cognitive intervention can play a positive role in delaying the process or even reducing the cognitive decline for both healthy controls [3] and amnestic mild cognitive impairment (aMCI) patients [4]. In particular, aMCI is a vital prodromal state of AD harboring memory impairment and has a high risk to progress into AD [5]. Multi-domain interventions targeting memory and non-memory domains simultaneously are urgently needed for an optimal aMCI intervention effect. However, most of the previous multi-domain cognitive intervention studies, e.g., the Finnish Geriatric Intervention Study to Prevent Cognitive Impairment and Disability (FINGER) [6], the French Multidomain Alzheimer Preventive Trial (MAPT) [7], the Dutch Prevention of Dementia by Intensive Vascular Care (Pre-DIVA) [8], the Drug and Alcohol Intervention Service for Youth (DAISY) [9], etc., evaluated the intervention outcomes based solely on neuropsychological assessment or simple characterization of brain anatomical structural changes (e.g., gray matter volume, cerebral ventricle volume) [10]. There is a critical need to develop a patient-specific *quantitative* analysis for the underlying *functional* brain regional activity changes during the multi-domain cognitive intervention process. Functional brain network analysis for AD intervention studies can offer great qualitative and quantitative insights into the brain micro-circuits alterations for MCI patients, and could also play an important role in the accurate prediction of the AD progression.

In this work, we develop and apply a new method based on an unsupervised Gaussian embedding-based functional brain network analysis for resting state fMRI data. Graph embedding methods have gained a lot of attention in recent years since they can effectively project large-scale networks to a low-dimensional latent space, while preserving the intrinsic network topological properties. The obtained graph embedding can be used for downstream graph processing tasks—such as link prediction, node classification, and community detection—much more effectively, easily and with high computational efficiency compared to other more classical methods.

Recent survey studies [11, 12] divided graph embedding techniques into three main categories: (1) matrix factorization-based approaches, (2) random-walk based approaches, and (3) deep learning-based approaches. For graph embedding methods, the primary challenge is to preserve the first-order and high-order proximity during graph embedding implementations. In order to tackle this problem, matrix factorization-based methods (e.g., GraRep [13] and HOPE [14]) construct a high-order proximity matrix based on transition probabilities and factorize it to obtain the node embeddings, but they are not easy to scale up for large network embeddings. Random walk-based methods (e.g., node2vec [15] and DeepWalk [16]) utilize different node neighbor set searching strategies through modified random walk paths to capture global and local proximities during the low dimensional point-vector embedding procedure. However, the major drawback of the aforementioned embedding approaches is the lack of capturing important “uncertainty” information for each node in the complex networks. Nodes with low degree contain fewer network connections, hence they have larger uncertainty than other nodes (or “entities” in the knowledge graph). Similarly, edges (relations) that link to more entities have a relative larger uncertainty than others. For the purpose of estimating uncertainty information for each node in the network, Gaussian embedding is uniquely positioned to derive and characterize embeddings in terms of means and variances in a space of multivariate Gaussian distributions. For example, Vilnis et al. [17] first proposed the word2Gauss method, which applied Gaussian embedding to map word types into a space of Gaussian distributions in order to model the uncertainty, entailment, and inclusion information of different word types in latent space. He et al. [18] proposed KG2G learning Gaussian embedding for latent knowledge graph representation. Moreover, Zhu et al. [19] proposed to apply deep variational network models in conjunction with the Wasserstein-2 (W2) distance and build a hybrid loss function to obtain Gaussian embeddings that preserve the transitivity in embedding space. Bojchevski et al. [20] employed a deep neural network model to learn node embedding as Gaussian distributions much more efficiently and robustly in latent embedding space for attributed and directed graphs. In particular, they developed an efficient way of predicting the effective dimensionality of the low-dimensional space (latent dimension) by monitoring during training the most “uncertain directions”, which are unstable and do not contribute to the low-dimensional embedded graph.

Compared with traditional graph embedding methods that project nodes into low-dimensional point vectors, deep neural network-based Gaussian embedding models, such as the Graph2Gauss model [20] can offer a very promising and novel approach for learning graph node representations (or “encodings”) as a latent space of Gaussian distributions in an inductive and unsupervised manner. In the latent graph embedding space, each node is encoded as Gaussian distributions with two different learned vectors (mean and variance). The mean vector reflects the position of the node while the variance, usually constructed in two different shapes (diagonal or spherical), provides important uncertainty information. Specifically, the learned uncertainty provides information on two critical aspects: 1) correlation with neighborhood diversity, i.e., larger variance reveals more diversity in the node’s k-hop neighborhood, 2) ability to discover the intrinsic latent dimensionality of the complex graph, which is close to the number of ground-truth communities in the graph.

In the present study, we focused on functional brain network analysis for aMCI patients, who completed a multi-domain cognitive training (MDCT) intervention that was designed at the PKU-sixth hospital of China. For each of 12 patients, resting-state functional MRI scans and cognitive assessment scores (MMSE [21] and MOCA [22]) were collected before and immediately after a 12-week intervention [4]. The new method we propose enables mapping of brain networks into multivariate probabilistic Gaussian distributions so as to detect the underlying link changes of functional brain connectomes after the MDCT intervention. Moreover, it provides *uncertainty estimation* for each node in the latent brain network representational space by performing deep learning-based Gaussian embedding for the weighted brain network computed from pre-processed fMRI data using the functional brain template [23]. We compared the new method against other methods, e.g., *node2vec* [15]. Most of the existing graph embedding methods focused only on a single and binary graph embedding. However, the human brain network is in the form of a weighted graph. Moreover, presently few works consider Gaussian embedding for multiple graphs, yet it is prerequisite for quantitative analysis of multi-subject brain networks before and after MDCT intervention. Hence, in our study, we propose a *multi-graph Gaussian embedding* (MG2G) method for the MDCT intervention dataset of aMCI patients (more details on MG2G are presented in Material and Methods and also in S1 Fig in S1 Appendix of Supporting Information).

## Materials and methods

### Ethics statement

The present study was approved by the ethics committee of Peking University Institute of Mental Health (Sixth Hospital), Beijing, China. All participants were fully informed regarding the study protocol and provided written informed consent.

### Participants

All aMCI participants were recruited from the Dementia Care and Research Center of Peking University Institute of Mental Health (DCRC-PKUIMH) between May 2015 and September 2015. Twelve of them met the inclusion criteria of MCI (stated below) and completed both a standardized neuropsychological evaluation and MRI scanning at Peking University Third Hospital. All participants were required to be 55 years old and above, right handed, and have an education level of no less than five years. The diagnosis of MCI was made according to Petersen et al. [24] as follows: (a) subjective memory complaint, confirmed by an informant; (b) a mini-mental state examination (MMSE) score of no less than 24; (c) an ADL score of no more than 26, and not diagnosed as having dementia (according to ICD-10 and NINCDS-ADRDA criteria). Other inclusion criteria were: a global clinical dementia rating score of 0.5 and no depressive symptoms (Hamilton Depression Scale score ≤ 12). Exclusion criteria were: a current or past neurological disorder or a current neuro-psychiatric disorder listed in the DSM-IV affecting cognition; currently taking cognitive enhancers; and any physical condition that could preclude regular participation in the intervention program.

### MDCT intervention and cognitive assessment

We used a self-controlled design to investigate the effect of the MDCT program on spontaneous brain activity in older participants with aMCI. Every patient underwent 24 training sessions delivered twice per week over approximately 12 weeks. Each session lasted 60 minutes and included tasks that covered three different cognitive domains. The participants spent 20 minutes engaged in each task per session. The 24-session intervention targeted multiple cognitive domains across the different sessions, including *reasoning, memory, visuo-spatial skill, language, calculation, and attention*. Neuropsychological assessments and MRI scans were conducted before and after the 12-week training program; details are described below.

#### Imaging protocol

MRI was performed using a 3T General Electric MRI 750 (Chicago, Illinois, United States) with an 8-channel sensitivity-encoding head coil (SENSE factor = 2.4), with parallel imaging using a Gradient-Recalled Echo-Planar Imaging (GRE-EPI), at the Peking University Third Hospital Neuroimaging Center. Two resting state BOLD fMRI imaging data were collected for each of the 12 aMCI patients, one before and one after MDCT intervention. The resting state functional MRI (rs-fMRI) data in each patient consisted of 230 functional volumes, each slice had a 64 × 64 grid, time repletion (TR) = 2000 ms, time echo (TE) = 20 ms, flip angle = 90°, field of view (FOV) = 240 × 240 mm2, 41 axial slices, thickness = 3.0 mm, and spacing between slices = 3.3 mm.

#### Cognitive assessment

We applied a comprehensive cognitive test battery to evaluate the cognition of patients at the baseline and after 12-week MDCT intervention. Global cognition was assessed via the MMSE (range 0–30) and MOCA (Montreal Cognitive Assessment) (range 0–30), with higher MMSE scores indicating higher levels of global cognition in both tests. Memory was evaluated via the Hopkins Verbal Learning Test-Revised, with higher MOCA scores indicating greater levels of memory (range 0–12). The speed of processing was examined using the Trail Making Test A, with lower scores indicating greater levels of processing speed. Visuo-spatial ability was examined using the Brief Visuospatial Memory Test-Revised, with higher scores indicating greater levels of visuo-spatial ability (range 0–12). Language function was examined using a verbal fluency test for animal naming, where higher scores indicate greater levels of language. Executive function was assessed via subtests, including a 100-stimulus version of the Stroop color and word test, a digit span test, a space span test, and a picture completion test; higher scores indicate greater levels of executive function. The complete cognitive assessment took about 120 minutes and took place at DCRC-PKUIMH.

### fMRI data pre-processing

The pre-processing of resting state fMRI data was carried out using Statistical Parametric Mapping (SPM12) [25] and Data Processing Assistant for the R-fMRI (DPARSF) toolkit [26]. The main steps included: (1) dropping off the first ten EPI volumes; (2) temporal correction for slice acquisition; (3) spatial normalization into the MNI space based on transformation parameters derived from aligning T1 images to the MNI standard template using diffeomorphic anatomical registration through the exponentiated lie algebra (DARTEL) method; (4) resampling to 3-mm isotropic voxels and spatially smoothing with a 4 mm full width at half maximum Gaussian kernel; (5) regressing out the following nuisances from each voxel’s time series, including 24 head motion parameters, global signal, cerebrospinal fluid, and white matter time series and linear trend; (6) filtering the residual time series within a frequency range of 0.01–0.1 Hz for reducing the effect of low-frequency drifts and high-frequency noise.

### Functional brain network construction

**Fig 1:**
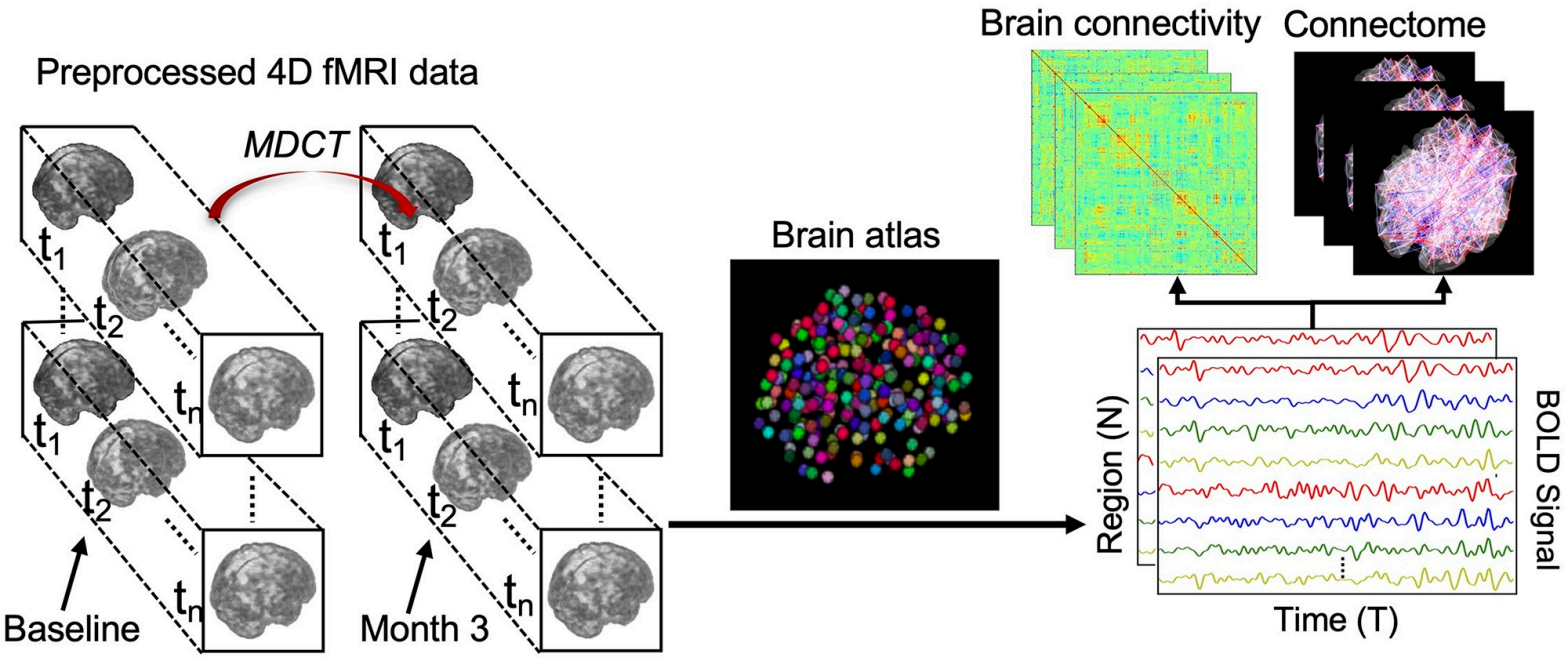
Illustration of brain connectivity construction workflow based on the functional brain atlas [13].

**Fig 2:**
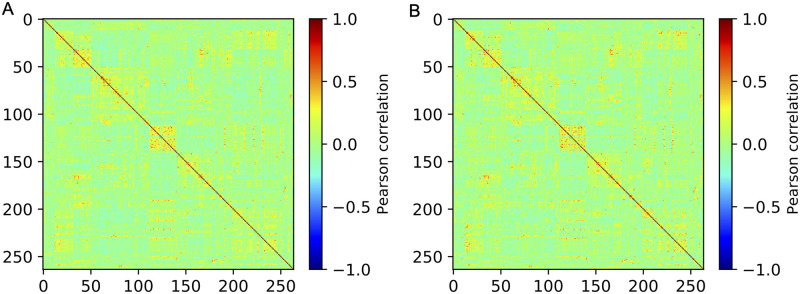
Functional brain connectivity matrices averaged across all 12 patients measured using the Pearson correlation. (A) Average brain connectivity at baseline. (B) Average brain connectivity after 12-week MDCT intervention. In (A) and (B), the X/Y axes represent the brain region indices of 264 brain regions defined in the brain atlas [13].

Based on the pre-processed fMRI images, the overall functional brain connectivity construction process is shown in Fig 1. First, we used a sphere-based functional brain atlas (Power et al. [23], 2011) to define 264 brain regions of interest (ROI) belonging to 14 communities (neural systems) in total. Then, the mean signals (time-series) were computed within spheres of fixed radius r (r = 5) around a sequence of voxels in T functional brain scans (T = 230). Finally, we computed the Pearson’s correlation coefficient across all pairs of time series to construct the brain connectivity matrix C and obtained the corresponding 3D visualizations for functional brain connectomes. Here, we did not pursue any corrections due to autocorrelation of the individual brain regions as the Durbin-Watson (DW) statistic we computed from our data has similar values as in the work of Arbabsbirani et al. [27], who have found no impact of the autocorrelation in functional connectivity in their study with fMRI data for schizophrenic patients. For every patient in the pre-processed fMRI dataset, we computed the brain connectivity matrices for fMRI data obtained at baseline (week 0) and week 12. The averaged brain connectivity results before and after interventions are shown in Fig 2.

### Multi-Graph2Gauss embedding approach for functional brain network analysis

**Fig 3:**
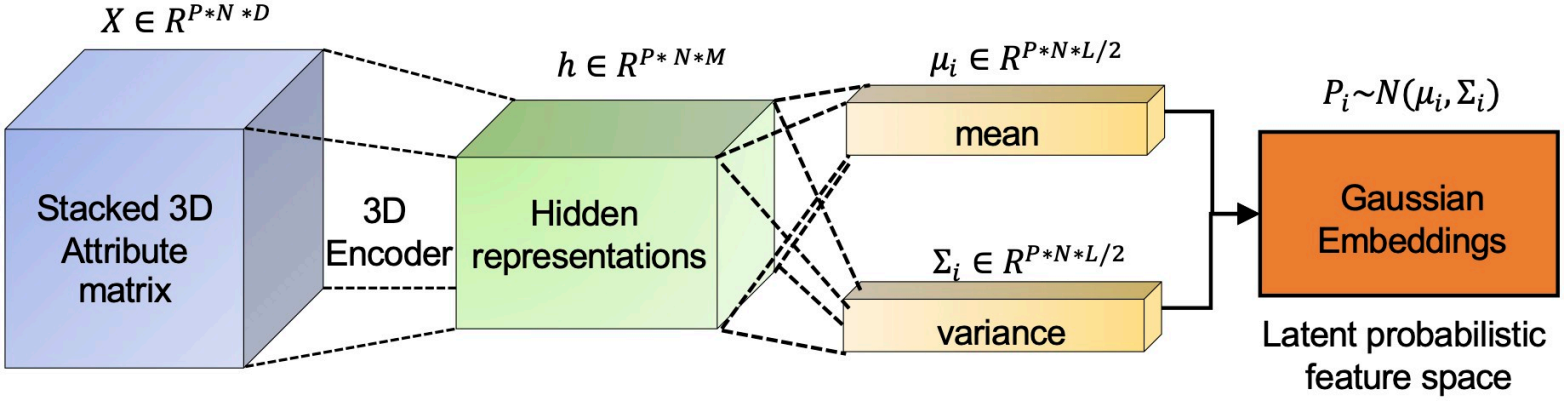
Main architecture of the proposed *MG2G* model for multiple human brain networks.

Since functional brain networks calculated from our raw fMRI data were undirected and weighted, in our work we extended the Graph2Gauss method [20] to a multi-graph Gaussian embedding (MG2G) prediction model and applied it for solving the functional brain network analysis problems in cognitive training evaluation of aMCI patients. The main purpose of the MG2G model is to learn useful low-dimensional probabilistic graph embeddings for multiple brain networks in complex high dimensional space to a uniform latent multivariate Gaussian distribution space, such that the model can effectively learn inherent stochastic embeddings through encoding both graph structure properties and node attributes from the original space. The obtained embeddings can be readily and efficiently used for downstream functional brain network analysis w.r.t. diverse brain disorders. The specific schematic illustrating the MG2G method for function brain network representation learning with a 3D neural network-based encoder can be seen in Fig 3.

Before we start to encode the brain networks, we first generate the node context (or neighbors) and node attributes based on the adjacency matrix for each brain network. In particular, for each of the *P* = 24 original brain connectivity matrices (*C*_*i*_, *i* = 1, 2, ‥, *P*), we obtained a thresholded adjacency matrix $A\in R^{N\times N}$ (*N* = 264 is the number of brain regions) by setting to 0 connectivity values below an empirical threshold (t = 0.1). We also tested the sensitivity of the threshold used here by setting the value to t = 0 so that we keep more links in the brain networks. However, we found almost identical results for the two threshold values, hence the link prediction performance is robust. Moreover, a value of *t* = 0.1 accelerated the training process. In order to capture both local and global graph structure properties during graph embedding, we employed the weighted-based *k*-hop neighborhood sampling method to measure the node similarity and sample the corresponding node context (or neighbors) for every node in the brain networks. Specifically, we computed the shortest distances between node pairs based on the weighted adjacency matrices and generated k-hop neighborhoods (*N*_*ik*_, *k* = 2, 3) using thresholds for the shortest distances. Moreover, node triplet sets (*D*_*t*_) (see Eq. (3) in the S1 Appendix) were generated based on the hops extracted from different brain networks, which was subsequently used for model training and optimization. In addition, for each node i, we assigned as node attributes the *i*^*th*^ row vector of *A*. In other words, each node had as attributes the connectivity profile (connection weights) across all the *N* nodes in the network. Consequently, brain networks had *N*-dimensional node attributes, with the aim of subsequently compressing them to *L* dimensions via graph embedding.

Next, we elaborate on the method and provide some implementation details with reference to Fig 3 and also to S1 Fig in the S1 Appendix. In order to encode multiple graph data jointly into the same space, our model takes as an input the computed undirected and weighted functional brain networks represented by the *attribute matrices* $X={\left\{X_{p}\right\}}_{p=1}^{P},X\in R^{P\times N\times D}$. Here, *N* is the number of brain regions, *D* is the number of attributes, which we take here equal to *N* as explained above, and *P* is the number of subjects. We use a 3D encoder to encode node attributes *X* into intermediate hidden representations. The hidden representation is realized through a sequence of hidden layers, i.e.,

$$\begin{array}{c} h_{i}^{k}=ReLU\left(h_{i}^{k-1}W_{i}^{k}+b^{k}\right),W_{i}^{k}\in R^{N\times M},b_{i}^{k}\in R^{M}, \end{array}$$

where *k* denotes the index of hidden layer and *M* is the dimension of the hidden representation that is smaller than the attribute dimension (*D*). Here, we used a single hidden layer (*k* = 1) of size *M* = 128 in our 3D model implementation. The outputs of MG2G are node-wise low-dimensional multivariate Gaussian distributions $P_{i}=N\left(\mu_{i},\Sigma_{i}\right),i=1,2,\ldots ,N$ parameterized by the mean vector *μ*_*i*_ and the covariance *Σ*_*i*_, where

$$\begin{array}{c} \mu_{i}=h_{i}^{k}W_{\mu }+b_{\mu }),W_{\mu }\in R^{N\times L/2},b_{\mu }\in R^{L/2}; \end{array}$$

the covariance matrix Σ_*i*_ is defined as a square matrix with variance *σ*_*i*_ as its diagonal elements, where

$$\begin{array}{c} \sigma_{i}=elu\left(h_{i}^{k}W_{\sigma }+b_{\sigma }\right))+1,W_{\sigma }\in R^{N\times L/2},b_{\sigma }\in R^{L/2}, \end{array}$$

where *elu* is the activation function. Finally, all the parameters of our model including weights ($W_{i}^{k},W_{\mu },W_{\sigma }$) and biases (*b*^*k*^, *b*_*μ*_, *b*_*σ*_) are learned by minimizing the square-exponential loss function

$$\begin{array}{c} L=\sum [E_{pos}^{2}+exp^{-E_{neg})}], \end{array}$$

where $E_{pos}$ and $E_{neg}$ refer to the Kullback–Leibler (KL) divergence between the Gaussian embeddings of positive node pairs and negative node pairs in the node triplet set (*D*_*t*_), respectively. Lastly, the neural network was optimized by using the Adam algorithm in TensorFlow 1.14.0 with initial learning rate = 1e-3, maximum number of epochs = 1000 and number of hidden units = 128.

In comparison with the basic principles of *Graph2Gauss* summarized in S1 Appendix of Supporting Information, our MG2G model for functional brain networks made three contributions: i) we made use of weighted (as opposed to binary) *symmetric adjacency matrix* to compute k-hop neighbors and triplet sets; ii) we added the connection weights as edge attributes to provide extra information for graph embedding, and iii) we extended the method to multiple graph data. As a metric of comparison and to capture the subtle differences before and after MDCT intervention, we made use of the Wasserstein-2 distance in Eq (1) for quantitative evaluation of ROI-specific changes between encoded probabilistic Gaussian distributions with respect to each patient‘s brain networks before and after interventions.

$$\begin{array}{c} W2{\left(P_{i},P_{j}\right)}^{2}=W2{\left(N\left(\mu_{i},\Sigma_{i}\right),N\left(\mu_{j},\Sigma_{j}\right)\right)}^{2}=\|\mu_{i}-\mu_{j}{\|}_{2}^{2}+\|\Sigma_{i}^{1/2}-\Sigma_{j}^{1/2}{\|}_{F}^{2}. \end{array}$$ (1)

## Results

### Graph embedding model training and evaluation

**Fig 4:**
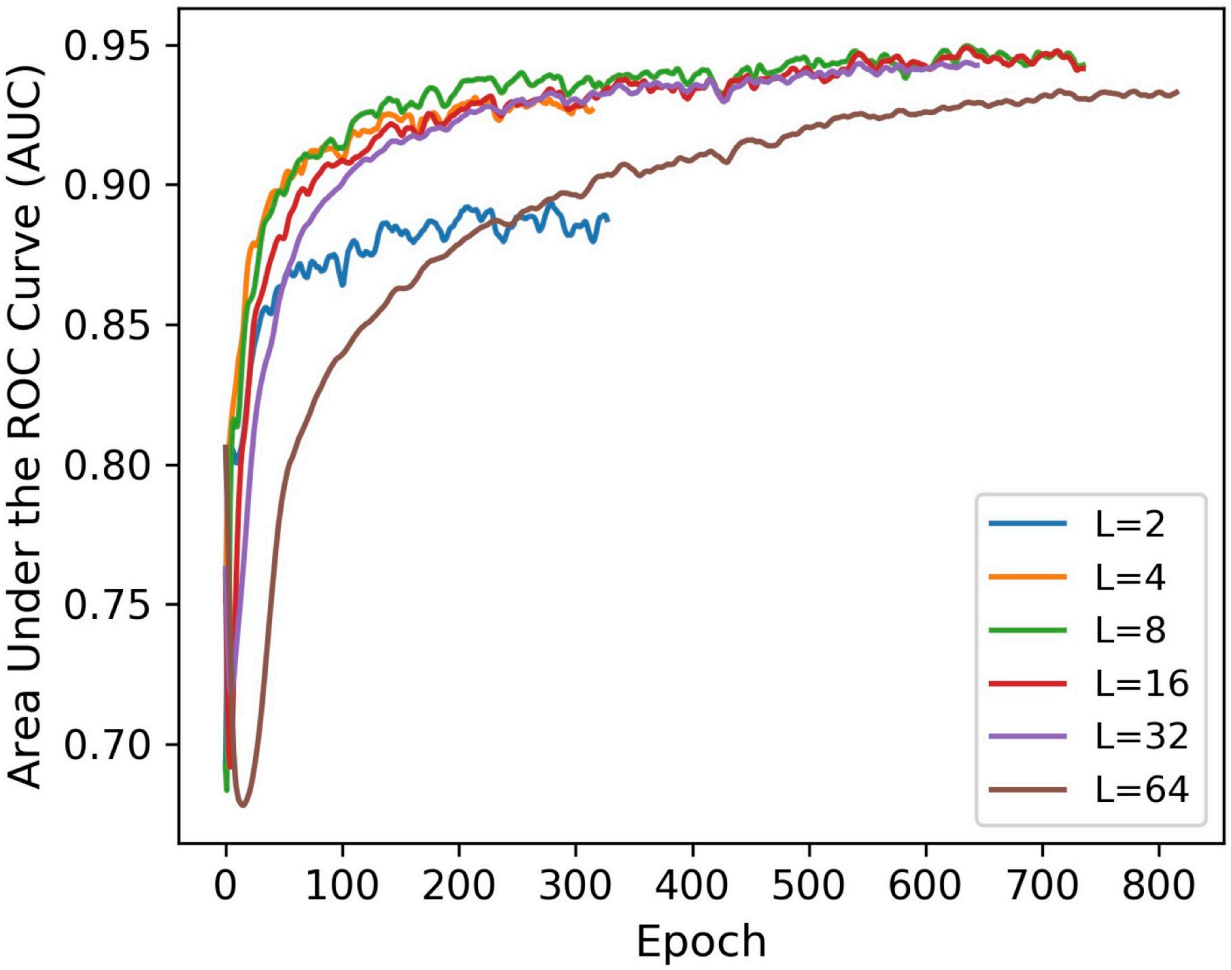
MG2G model performance in link prediction for different values of embedding size (L). Results are shown for the validation dataset based on *L* = 2, 4, 8, 16, and 32, with *K* = 2 (*k*-hop neighborhoods).

An important application of graph embedding is *link prediction* that quantifies how well a model can predict unobserved edges. In order to evaluate the representational performance of the MG2G method, we carried out link prediction experiments on brain networks computed from resting state fMRI data recorded from 12 aMCI patients before and after MDCT intervention. The brain networks were constructed by computing the Pearson’s correlation coefficient between the fMRI time series of 264 brain regions of interest (ROI) that belong to 14 communities (neural systems) according to the Power et al., 2011 brain atlas [23]. We split the total edges obtained from the network adjacency matrices into three sets: a training set (85%), a validation set (10%) and a test set (5%). The performance in the validation set in terms of AUC (area under the ROC curve) for different values of embedding size *L* is shown in Fig 4 for a fixed value of K = 2; here K denotes the maximum distance we consider for finding the k-hop neighborhoods.

**Fig 5:**
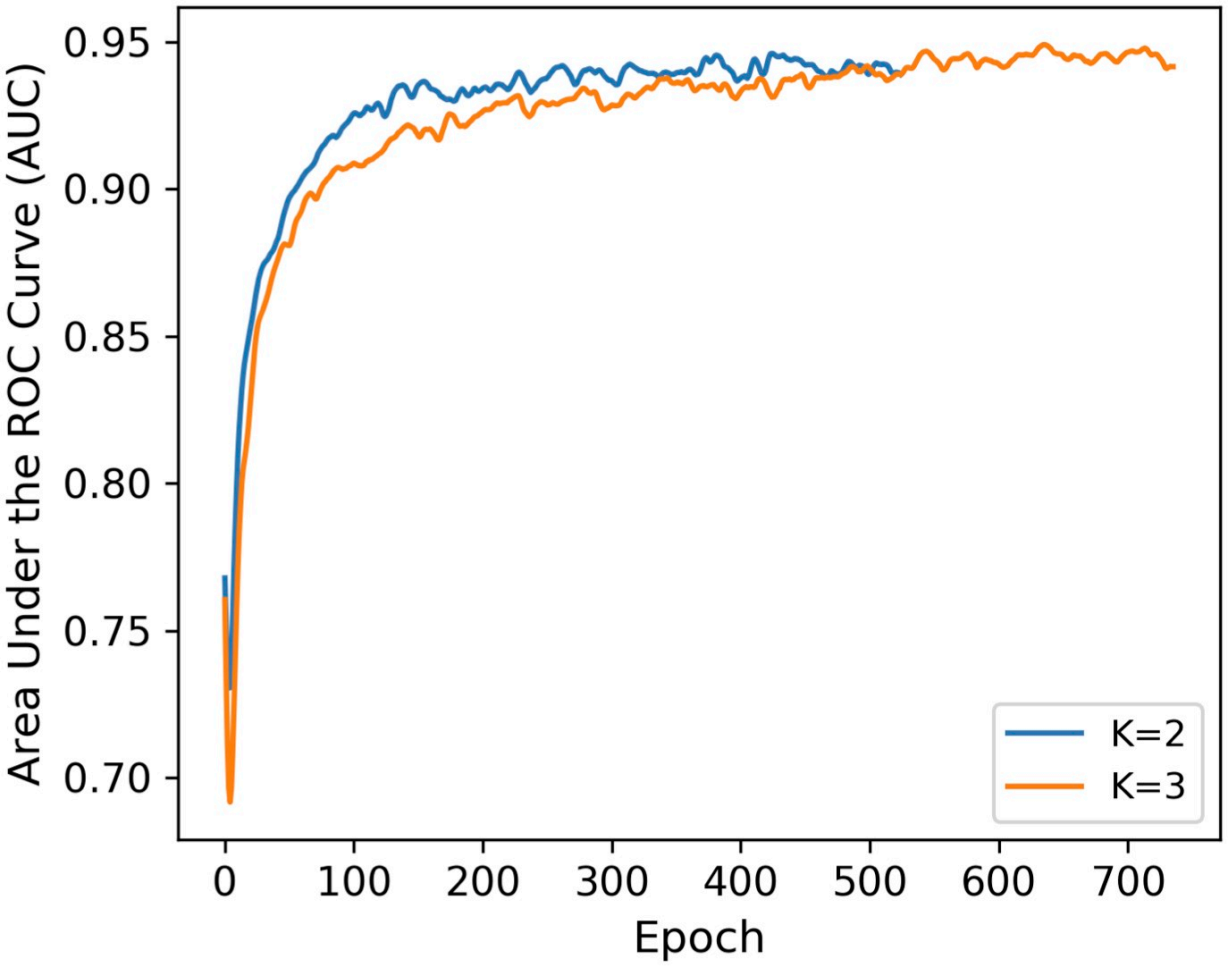
Evaluation of link prediction performance using MG2G based on different k-hop neighborhoods. AUC values vs. number of epochs based on *k* = 2 (blue curve) and *k* = 3 (yellow curve); the embedding size (*L*) was equal to 16.

MG2G achieved high AUC performance in link prediction for embedding size *L* equal to 4, 8, and 16. In contrast, the AUC performance was low for *L* = 2 because embeddings with small size cannot sufficiently capture the representational information of the original graph data. Performance was also low when *L* increased to 32 because an embedding size larger than the latent dimension of the graph may include higher levels of noise. High values of *L* are also not desirable because they increase computational cost. In addition to evaluating the sensitivity to different embedding size values (*L*) in link prediction, we also evaluated the performance of MG2G for different k-hop neighborhoods (*K* = 2 vs. *K* = 3); the results are shown in the Fig 5 indicating that *K* = 2 is adequate. Finally, for the test set we obtained AUC value of 0.945 in link prediction for a fixed embedding size *L* = 16 and k-hop neighborhood *K* = 2.

### Quantification of intervention-related brain network alterations using the Wasserstein distance

By performing Graph Gaussian embedding for all patients’ brain networks, every brain region (node) is represented by multivariate Gaussian distributions in a latent space. In order to assess the complex functional network alteration patterns within each patient, we quantified how each node moved in the latent space following the intervention. Specifically, we measured the distances of *each patient’s* brain network embeddings (or Gaussian distributions) for each ROI before and after intervention. The distance measure relied on the Wasserstein-2 distance (W2), which quantifies distances between Gaussian probability distributions. Since our dataset lacks a control group and W2-distance is positive without a known parametric distribution, there is no obvious parametric or non-parametric statistical procedure to apply to these results. However, in the next section we will provide largely consistent results with an alternate group-level analysis.

**Fig 6:**
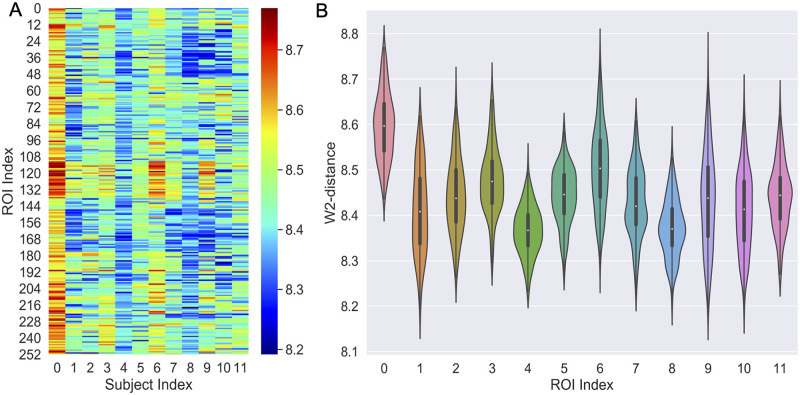
Within-subject intervention-related brain network alterations. (A) W2-distance before and after intervention for each of the 264 ROIs across the 12 patients (*L* = 16, *K* = 2). (B) Violin plots of the W2-distance distribution over the 264 regions for each of the 12 patients.

The within-subject W2 distances for each of the 12 patients are shown in Fig 6A, with the 264 ROIs and related 14 systems in the brain atlas [23] described in the Supplementary S2 Table. As a reference value, we computed the W2 distance among similar subjects before intervention, e.g., subject 4 and 5, who had similar MOCA and MMSE scores. We found a mean W2 value of 8.35, which is below the W2 distance values recorded in all subjects before and after intervention. We observe across different patients that the ROI IDs from 112 to 138 exhibited large variations before and after intervention among most patients, and most prominently for subject 3, 6 and 9. Based on the system information from S2 Table, these regions mainly fall into three functional systems: *default mode, memory retrieval, and visual systems*. Moreover, subject 0 had the greatest number of ROIs with large W2-distance between intervention, and we note that this patient was also diagnosed with depression symptom. There were also patients with smaller variations, namely subjects 4 and 8, compared to other aMCI patients after intervention.

To better assess the overall network alteration at the subject-level, we used a “violin plot” (combination of box-plot and density plot) to visualize the W2-distance distributions and probability densities for different patients (Fig 6B). Each “violin” contains a box-plot (white dot, vertical thick black box and thin black line). The white dot represents the median of W2 distances at each column in Fig 6A, the vertical thick black box indicates the inter-quartile range, and the thin black line denotes the extensions to the maximum and minimum values. The shaded areas surrounding the box plot show the probability density of the W2-distances across the 264 brain regions for each patient. These results reveal that patients varied considerably with respect to the network alterations, with some subjects exhibiting large W2 medians and variability (e.g., subjects 0 and 6) and others the opposite (e.g., subjects 4 and 8), while there are also some unique subjects with multi-modal shape of the W2 distribution (e.g., subject 7).

**Fig 7:**
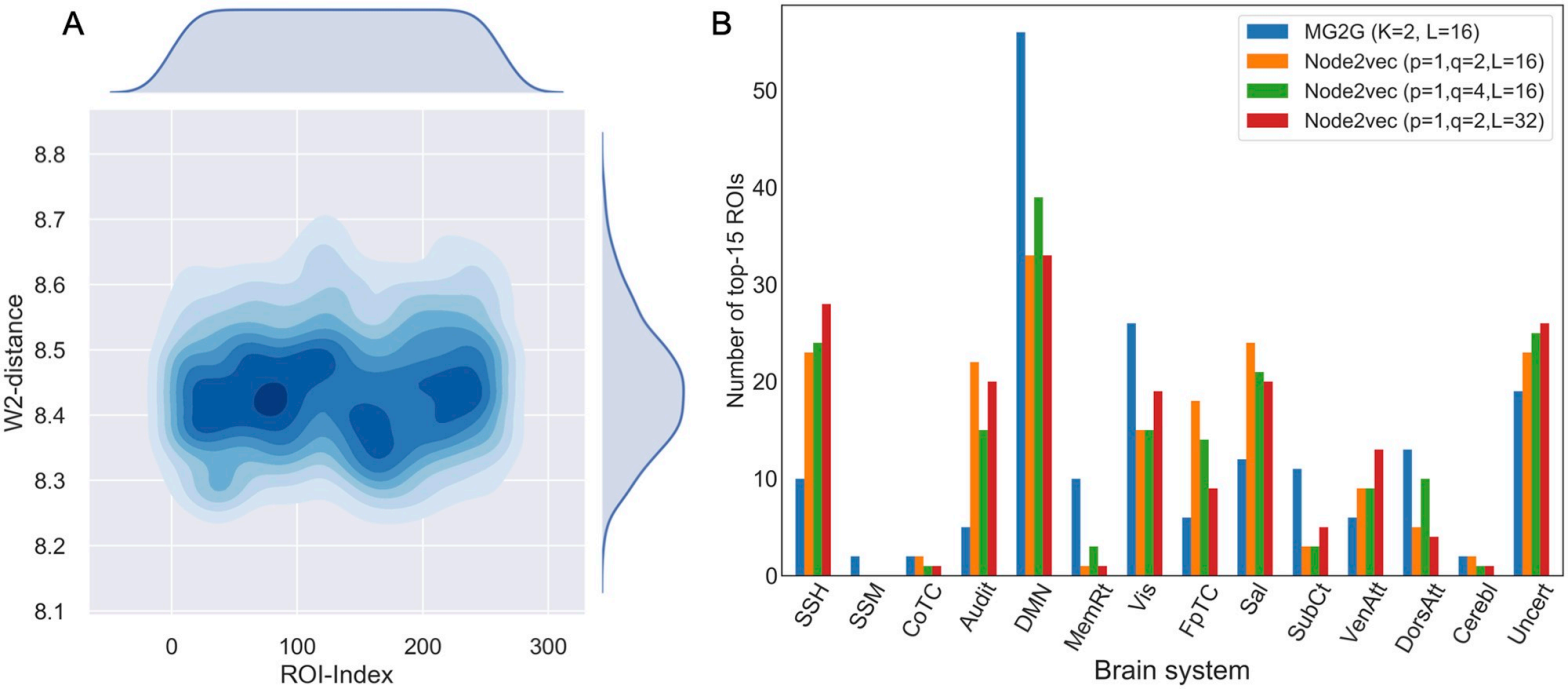
Within-subject intervention-related alterations at ROI-level and system-level. (A) Kernel Density Estimation plot of the W2-distance across all 264 ROIs. (B) Quantification of functional/system-level changes for all 12 patients before and after MDCT intervention based on MG2G (blue) and node2vec (yellow, green, and red, corresponding to different node2vec parameters). SSH: sensory/somatomotor hand; SSM: sensory/somatomotor mouth; CoTC: cingulo-opercular task control; Audit: auditory; DMN: default mode; MemRt: memory retrieval; Vis: visual; FpTC: fronto-parietal task control; Sal: salience; SubCt: subcortical; VenAtt: ventral attention; DorsAtt: dorsal attention; Cerebl: cerebellar; Uncert: uncertain.

To more specifically quantify the ROI-level W2-distance density changes across the 12 patients, we constructed the Kernel Density Estimation (KDE) plot in Fig 7A. The brain regions (ROIs) around the index ranges of 100-150 and 221-240 exhibited larger alterations (in terms of the W2-distance) compared to other brain regions. As shown in S2 Table, these regions belong to the following communities (brain systems): *default mode, memory retrieval, visual and dorsal attention*. Additionally, from the KDE plot we can also distinguish three dark blue areas (default mode, visual, frontal-parietal task control, dorsal attention and uncertain) with high probability densities of W2-distance compared to other regions. Subsequently, we obtained the top-15 brain regions for *all patients* measured by the W2-distance, and identified the brain systems they belong to, shown in blue bars in Fig 7B. Here, the vertical axis denotes the total number of top-15 ROIs corresponding to each community. The highest system-level MG2G results identified with this analysis were: *default mode, visual, uncertain, dorsal attention, salience, subcortical, sensory/somatomotor hand, and memory retrieval*, which overlap with the dark blue areas in Fig 7A.

To further validate these system-level results, we also performed a secondary analysis using a different graph-embedding method, the deterministic “node2vec” [16]. The *node2vec* results are shown in yellow, green and red bars in Fig 7B; here we performed a similar analysis as in MG2G, but the metric was Euclidean distance because *node2vec* is deterministic and nodes are mapped to point-vectors in the latent space. We assessed the sensitivity of the results change for different embedding size (*L* = 16, 32) and different hyperparameters (*p* and *q* values) in *node2vec*; these values control the neighborhood exploration in *node2vec*. The *default mode, sensory/somatomotor hand, auditory, visual, salience, and uncertain* communities exhibited large subject-level intervention effects. Additional system-level comparisons at the single-subject level can be found from S3 and S4 Figs in Supporting Information using both the proposed MG2G as well as the node2vec method. We observed some variability among the patients and the two methods (MG2G and node2vec) but overall the top-15 changes in brain regions among the 12 patients mostly occurred in the *default mode, visual, uncertain, salience, memory retrieval, fronto-parietal task control, dorsal attention*.

### Statistical evaluation of intervention-related brain network alterations at the group-level

Here we quantified the intervention-related brain network alterations by defining a new measure, the *reorganization index*, which captures cross-subject W2-distance intervention effects. For every pair of *different* subjects, we computed the W2-distance per ROI when: i) one subject was before and the other after intervention (between-pair), or ii) when both subjects were paired before intervention (within-pair). The former assessed cross-subject intervention-related effects, whereas the latter established a baseline cross-subject W2-distance. We then defined the reorganization index RI as the averaged W2-distance of the between- minus within-pairs. Given 12 patients, we obtained 66 between-pair W2-distances matched by an equal number of within-pair W2 distances, allowing us to perform one-sample t-tests for statistical evaluation.

The between-pair and within-pair distances are exemplified for the ROIs belonging to the “Sensory/Somatomotor Hand” neural system in S5 Fig of Supporting Information. The between-pair (blue) were largely above the within-pair (red) W2 distances, demonstrating that RI increased due to the intervention for most of the ROIs.

**Fig 8:**
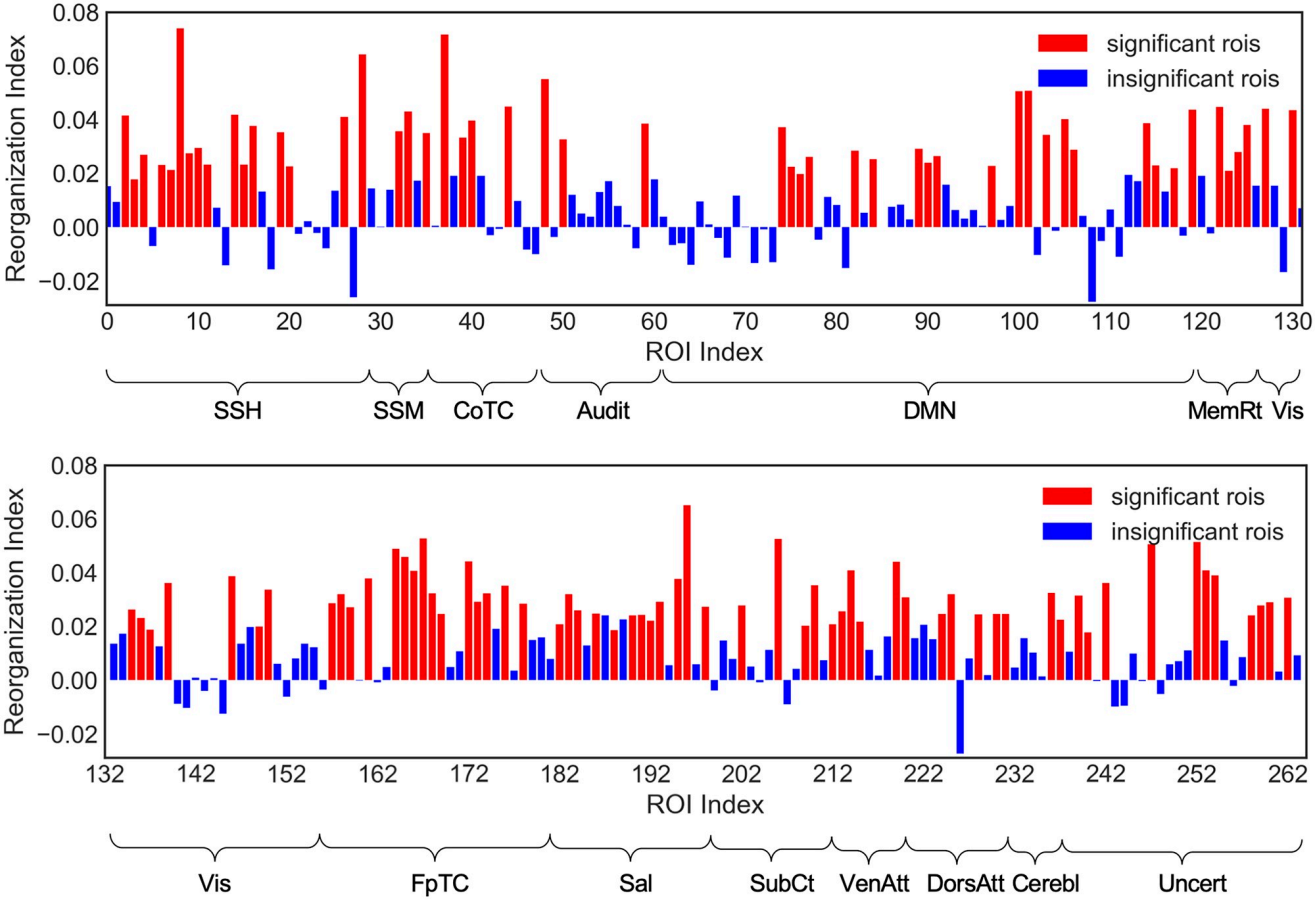
Reorganization index for each of the 264 ROIs. A large number of ROIs had significant RI (red bars; *p* < 0.05, FDR corrected), suggesting extensive intervention-related brain network reorganization. System name abbreviations same as in Fig 7.

**Fig 9:**
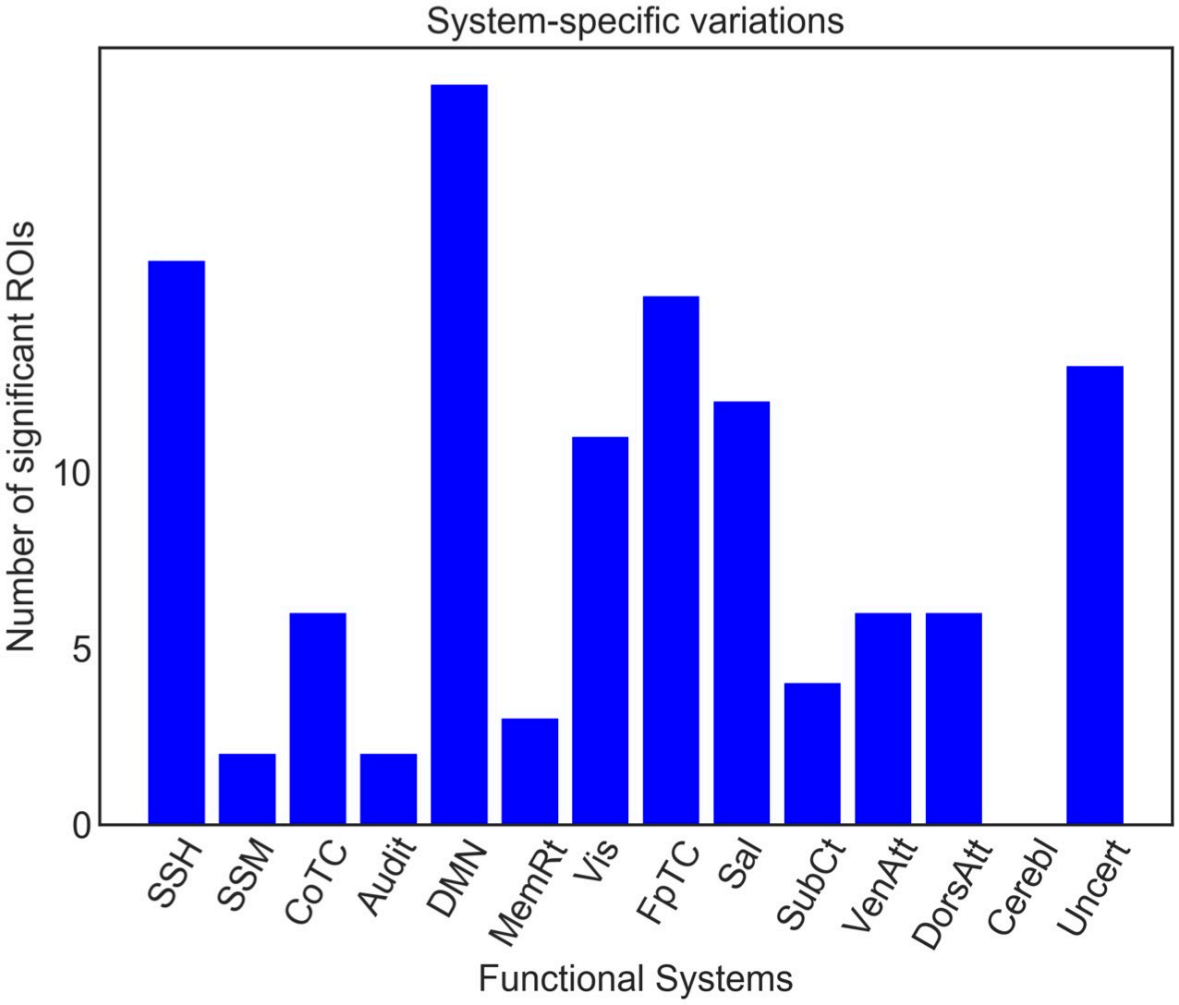
Number of ROIs with significant network alterations (significant RI index) contained within different functional brain systems. System name abbreviations same as in Fig 7.

RI results for all 264 ROIs are shown in Fig 8, with statistically significant results highlighted with red bars (*p* < 0.05, one-sample t-test, false discovery rate corrected). The majority of the ROIs had significantly positive RI, which suggests extensive fMRI brain network reorganization following the MDCT intervention, i.e., a positive RI value for a given brain region suggests network alterations due to the intervention. This is because the dots would tend to cluster in the same region in the embedded space for within-intervention subjects (both no intervention, or both intervention), but would map to distant areas for between-intervention subjects. More details can be found in S6 Fig. In Fig 9, we counted the number of significant ROIs belonging to each neural system. The results indicate that the most extensive brain network reorganization encompassed the *default mode, somatosensory/somatomotor hand, fronto-parietal task control, visual, salience, dorsal attention and uncertain* brain systems. These systems largely overlap with the neural systems identified with the within-subject analysis in the previous section. A list of the significant ROIs contained within each neural system is presented in Table 1.

**Table 1:** Names of ROIs with significant network alterations (significant RI index) for each brain system.

System	Significant ROI List (Quantity)
SSH	Inferior Parietal Lobule(1), Medial Frontal Gyrus(3), Paracentral Lobule(1), Postcentral Gyrus(6), Precentral Gyrus(4), undefined(1)
SSM	Precentral Gyrus(2)
CoTC	Cingulate Gyrus(1), Insula(2), Medial Frontal Gyrus(1), Middle Frontal Gyrus(1),Superior Temporal Gyrus(1)
Audit	Precentral Gyrus(1), Superior Temporal Gyrus(1)
DMN	Angular Gyrus(1), Anterior Cingulate(1), Cingulate Gyrus(1), Inferior Frontal Gyrus(1), Medial Frontal Gyrus(3), Middle Temporal Gyrus(6), Parahippocampa Gyrus(1), Posterior Cingulate(2), Precuneus(1), Superior Frontal Gyrus(4)
MemRt	Cingulate Gyrus(1), Precuneus(2)
Vis	Cuneus(3), Inferior Occipital Gyrus(2), Lingual Gyrus(2), Middle Occipital Gyrus(2), Parahippocampa Gyrus(1), Sub-Gyral(1)
FpTC	Inferior Frontal Gyrus(2), Inferior Parietal Lobule(4), Middle Frontal Gyrus(7), Middle Temporal Gyrus(1), Superior Parietal Lobule(1)
Sal	Anterior Cingulate(2), Cingulate Gyrus(1), Extra-Nuclear(1), Inferior Frontal Gyrus(1), Middle Frontal Gyrus(3), Sub-Gyral(1), Superior Frontal Gyrus(1), Supramarginal Gyrus(1), undefined(1)
SubCt	Extra-Nuclear(3), Thalamus(1)
VenAtt	Inferior Frontal Gyrus(2), Inferior Parietal Lobule(1), Superior Frontal Gyrus(1), Superior Temporal Gyrus(2)
DorsAtt	Middle Frontal Gyrus(2), Middle Temporal Gyrus(1), Sub-Gyral(1), Superior Parietal Lobule(2)
Uncert	Culmen(1), Fusiform Gyrus(1), Inferior Occipital Gyrus(2), Inferior Temporal Gyrus(2), Lingual Gyrus(2), Middle Frontal Gyrus(1), Sub-Gyral(1), Superior Frontal Gyrus(1), Uncus(2)

System name abbreviations same as in the S2 Table.

### Nodal uncertainty quantification

**Fig 10:**
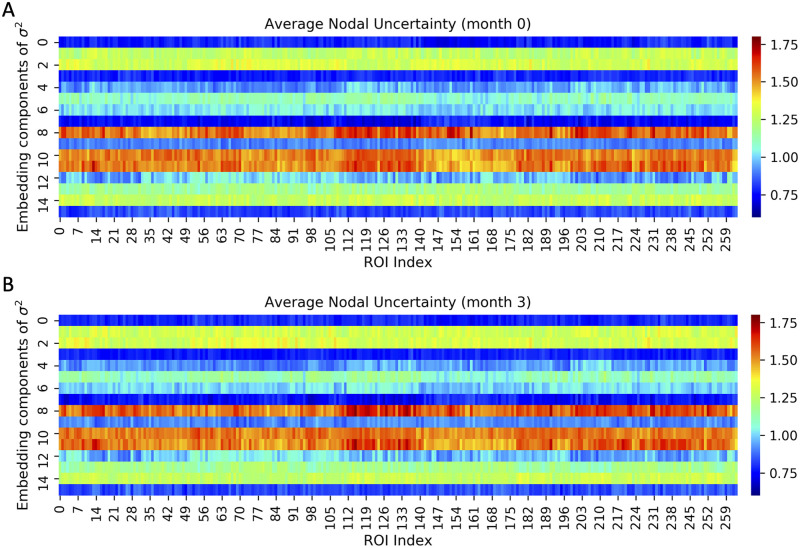
Uncertainty quantification using the MG2G approach. Average nodal uncertainty (variance-*σ*^2^) results for 12 patients before (A) and after intervention (B); (embedding size L = 16).

With Graph2Gauss embedding, every brain region was encoded as a multivariate Gaussian distribution. Hence uncertainty, quantified by the variance, can also be assessed using this graph embedding approach. Fig 10 illustrates the nodal uncertainty results of graph embedding at baseline and after intervention averaged across all patients. The vertical axis shows the embedding variance for each of the L = 16 dimensions. Dimensions 8, 10, and 11 had consistently high variance values for the majority of nodes before and after intervention. Dimensions with high uncertainty are unstable and do not contribute to a low-dimensional embedding in the latent space [20]. Thus, we can infer the effective latent dimension to represent our brain network to be equal to (L—3) by excluding the highly unstable dimensions. This yields an effective dimension of 13 (since the embedding dimension was L = 16), which is approximately equal with the ground truth community number (14) in the brain atlas. Therefore, our proposed method for fMRI data analysis not only predicted the latent representations, but also yielded the effective dimensionality of the low-dimensional space (latent dimension) by monitoring (during training) the “uncertain” dimensions. More detailed uncertainty quantification results by plotting the corresponding Gaussian distributions are shown in S2 Fig of Supporting Information.

## Discussion

The new method MG2G we introduced, and other recent graph embedding techniques, hold great promise in diverse real-world applications. However, so far, the studies incorporating prevalent graph embedding techniques for the analysis of complex and heterogeneous *functional brain network* systems for brain disorders (e.g. Alzheimer’s, Parkinson’s, etc.) are scarce. For example, Rosenthal et al. [28] first proposed to use a connectome embedding method, node2vec [15], for the mapping of high-order relations between brain structure and function. As discussed earlier, this method cannot model important uncertainty information about nodal embedding in the latent space. We have applied node2vec in our study to verify the results of MG2G, which in addition can effectively quantify uncertainty for the learned node representations. Therefore, *Gaussian embedding* can facilitate functional brain connectome analytics by employing a stochastic quantitative analysis, which is necessary given the lack of big data and the sensitivity and diversity of the human brain connectomes. To this end, we proposed a new functional brain network analysis framework based on multiple brain connectome Gaussian embeddings via deep neural networks, combined with weighted information of the original graphs. Additionally, we adopted the Wasserstein distance (*W2*) to quantify the brain region (ROI)-level differences between the multivariate embedded Gaussian distributions before and after intervention (Fig 6A). We constructed violin and KDE plots to estimate and display the W2 distance distributions (Figs 6B and 7A) from two different perspectives (patient-specific and ROI-specific) and developed a group-level analysis to statistically validate our findings (Figs 8 and 9). Our results demonstrated that Gaussian embedding-based functional brain network analysis can automatically and quantitatively detect the underlying multiscale (region → system → subject) *subtle changes* of brain networks after non-pharmacological MDCT interventions for aMCI patients. Moreover, we demonstrated two main advantages of the nodal embedding uncertainty in our study: i) we can obtain the intrinsic dimensionality (L) of the brain network, and ii) we can quantify the heterogeneity (diversity) of node’s neighbors. The latter is because the high uncertainty to some nodes is due to potential connections with neighbors of different communities with possibly contradicting underlying patterns.

Furthermore, the deep neural network-based model we employed in our study enabled learning the highly non-linear mapping from the original high-dimensional brain network space into low-dimensional Gaussian distributions, while at the same time quantifying the uncertainty about the node embeddings. This is in line with the recent successes of emerging deep learning techniques in diverse fields, when compared to traditional matrix-factorization methods (e.g. SVD [29]) and random walk-based models (e.g., node2vec [15]). Our MG2G model can readily scale up to large-scale network applications unlike traditional methods.

To evaluate the robustness and generalization of the MG2G method, we compared with the *node2vec* method employed in the work of Rosental et al. [28]. We compared the two methods (see S3 and S4 Figs in Supporting Information for details) using the same data as in our main study. Another alternative method is spectral embedding [30] designed to use an “informative” eigenvector decomposition, however, it becomes inefficient and unstable for large-scale and noisy fMRI data [31]. In contrast, the *node2vec* approach produced comparable results as our proposed MG2G method (see Fig 7B and in S3 and S4 Figs in Supporting Information) but ignored critical uncertainty information about the node embeddings and the intrinsic system dimensionality. Such information is potentially important for the dynamic, heterogeneous and complex functional role of different regions in the brain connectome. Our proposed deep neural network-based Gaussian embedding model can overcome the aforementioned problems effectively, and obtain *probabilistic* node representations, while preserving both local and global graph topology properties of brain networks. Furthermore, we also validated our method both with and without global signal regression, and the corresponding results are shown in S6 Appendix.

In addition to the within-subject analysis quantifying network alterations after intervention, we also analyzed statistically network alterations at the group-level by defining a new measure, the reorganization index (RI). In this case too, we found that a large number of ROIs were affected after intervention (Fig 8), and these changes at the system/community level (Fig 9) were comparable to the ones we obtained with the within-subject analysis in Fig 7B. Taken together, our results using two different approaches (MG2G and node2vec) and two different methods of analysis (top-15 ROI and t-test) showed consistency in the regions affected by the MDCT intervention, with details of each region presented in S2 Table in Supporting Information.

In addition to fMRI networks, in previous work [4] we have investigated the MDCT intervention effects on structural MRI data and found significant increases in gray matter volume in the right angular gyrus and other subareas following the MDCT intervention. In the current study, we further investigated the underlying MDCT intervention effects at both ROI-level and community-level on the fMRI networks. Therefore, MG2G can provide a more elaborate, cross-modality quantification of network alterations. Specifically, we quantified the differences between probabilistic Gaussian embeddings of functional brain connectomes before and after intervention using the W2-distance metric. The results revealed significant changes on an extensive number of brain regions (Fig 8 and Table 1). Also, system-level changes occurred primarily in the *default mode, somatosensory/somatomotor hand, fronto-parietal task control, memory retrieval, visual and dorsal attention* brain systems (Figs 7B and 9). Moreover, network alterations varied across patients (Fig 6), which is consistent with the heterogeneous clinical score profiles.

The broad intervention-related alterations on the intrinsic functional networks may reflect adaptive mechanisms of information integration among different functional systems over the whole brain, due to putative co-activation during the multi-domain training. A previous study that used only explicit-memory training has found increased activation and connectivity in distributed neural networks mediating explicit-memory functions [32]. Hence, an integrated cognitive training that targets more cognitive domains should stimulate more diverse distributed networks underlying multiple cognitive functions. A recent study using MDCT in a healthy older population has found increased functional connectivity within three higher cognitive networks that overlap with our current study: default mode, salience, and central executive network [3]. Therefore, our findings here suggest that widespread changes in functional connectivity induced by MDCT may be due to an enhanced restoration by functional reorganization that benefits brain cognition.

In the future, to better assess and validate the MG2G method on the MDCT intervention study, we plan to extend our method to process multi-modality data (fMRI, MRI, MEG, genetic, and PET) given the multifaceted nature of AD and high-order subgraph (or community) level organization pattern recognition [33]. Moreover, as more subjects enroll in the study and longitudinal data become available, we will better characterize the effectiveness of the MDCT intervention. Specifically, it is important to complete a longitudinal study that facilitates dynamic brain network fluctuation modeling during intervention (i.e., temporal and spatial patterns). Collecting data from a control group will also enable a direct comparison of network alterations across populations for a deeper understanding of the underlying mechanisms of the MDCT intervention.

## Supporting information

S1 Appendix Graph2Gauss embedding method. (DOCX)

S2 Appendix Example of uncertainty quantification. (DOCX)

S3 Appendix Comparison of MG2G versus node2vec. (DOCX)

S4 Appendix Example of reorganization index (RI) estimation. (DOCX)

S5 Appendix Interpretation of the reorganization index (RI) in the embedding latent space. (DOCX)

S6 Appendix Comparison with and without global signal regression. (DOCX)

S7 Appendix Brain atlas description. (DOCX)

S1 Fig Illustration of the main workflow for the Graph2Gauss embedding approach. (TIF)

S2 Fig Gaussian distributions obtained from MG2G for one patient. (TIF)

S3 Fig Quantification of system-level changes for all 12 patients before and after MDCT intervention based on MG2G. (TIF)

S4 Fig Quantification of system-level changes for all 12 patients before and after MDCT intervention based on different parameters of node2vec. (TIF)

S5 Fig Reorganization index results for 30 ROIs in the ‘somatosensory/somatomotor hand’ functional brain system. (TIF)

S6 Fig 2D visualization for different brain regions embeddings before and after intervention. (TIF)

S7 Fig W2 distance versus ROI for two randomly selected subjects based on fMRI brain networks, computed from time-series with (w/) and without (w/o) global signal regression. (TIF)

S1 Table Top 15 changing ROIs based on Gaussian embeddings learned from signals with and without global signal regression operation. (DOCX)

S2 Table Functional brain atlas information. (DOCX)
