# Supplementary material for: A new Graph Gaussian embedding method for analyzing the effects of cognitive training
Source: PLoS Comput Biol. 2020 Sep 17;16(9):e1008186. doi: 10.1371/journal.pcbi.1008186 (PMC7524000; doi:10.1371/journal.pcbi.1008186)
Supplement: S1 Appendix — (DOCX) [file pcbi.1008186.s001.docx]

Supporting Information for

**A new Graph Gaussian embedding method for analyzing the effects of cognitive training**

Mengjia Xu^1,2^, Zhijiang Wang^3,4,5,6^, Haifeng Zhang^3,4,5^, Dimitrios Pantazis^2^, Huali Wang^3,4,5^*, Quanzheng Li^6^*

* Corresponding author. E-mail: li.quanzheng@mgh.harvard.edu (Q.L.); huali_wang@bjmu.edu.cn (H.W.)

# S1 Appendix. Graph2Gauss embedding method

The graph embedding problem can be interpreted mathematically as follows. Given a directed/undirected graph *G* = (*A, X*) with *V* and *E* the corresponding vertex and edge sets, where *A* denotes the (symmetric or asymmetric) adjacency matrix of size $N\times N$, and *X* is the attribute matrix of size $N\times D$, the aim of Graph2Gauss is to project every node from a high-dimensional space into a latent space of multivariate Gaussian distributions, while preserving the graph structure property. Nodes that are “close” in the original space will be mapped into latent space with similar node embeddings. For instance, the embedding of node *i* ($P_{i}$) can be represented by a *L*-dimensional Gaussian distribution with a mean vector ($\mu_{i}$) and a covariance (diagonal) matrix ($\Sigma_{i}$), where $L\ll D.$

$P_{i}\mathcal{\sim N}\left( \mu_{i}, \Sigma_{i} \right) \mu_{i}\in\mathcal{R}^{L}, \Sigma_{i}\in\mathcal{R}^{L\times L}$. (1)

In order to obtain the latent Gaussian representation inductively for every node in a graph, Graph2Gauss model consists of four main parts: i) Valid node triplet ($D_{t}$) generation based on the adjacency matrix ($A$) using the k-hop neighborhood ($N_{ik}$) sampling technique for preserving the multiscale graph structure property, see details in Eqs. (2-3). ii) Unsupervised low-dimensional node embedding learning using a deep encoder incorporating node attributes ($X$), such that each node in the high-dimensional space is encoded as a low-dimensional *multivariate Gaussian distribution* ($P_{i}\mathcal{\sim N}\left( \mu_{i}, \Sigma_{i} \right), i\in V$). iii) Energy estimation between Gaussian embeddings of positive and negative node pairs extracted from the node triplets. iv) Optimization of the Graph2Gauss model based on the *energy-based ranking* loss ($\mathcal{L}$), *i.e.,* learning the hyper-parameters ($W_{k}, W_{h}, W_{\mu}, W_{\Sigma}, b_{k}, b_{h}, b_{\mu}, b_{\Sigma}$) of the Graph2Gauss model through minimizing the “*square-exponential* loss” denoted in Eq. (6) below. This will ensure that the learned optimal node embeddings satisfy the energy ranking constraints (see Eq. (4)) that the energy between embeddings of positive node pairs ($E_{pos}$) is lower than the energy computed for the embeddings of negative node pairs ($E_{neg}$). Here, the embedding energy between node pairs is represented by the asymmetric *KL-divergence* measure (see Eq. (5)). The complete workflow of Graph2Gauss is illustrated in S[1](#_30j0zll) Fig.


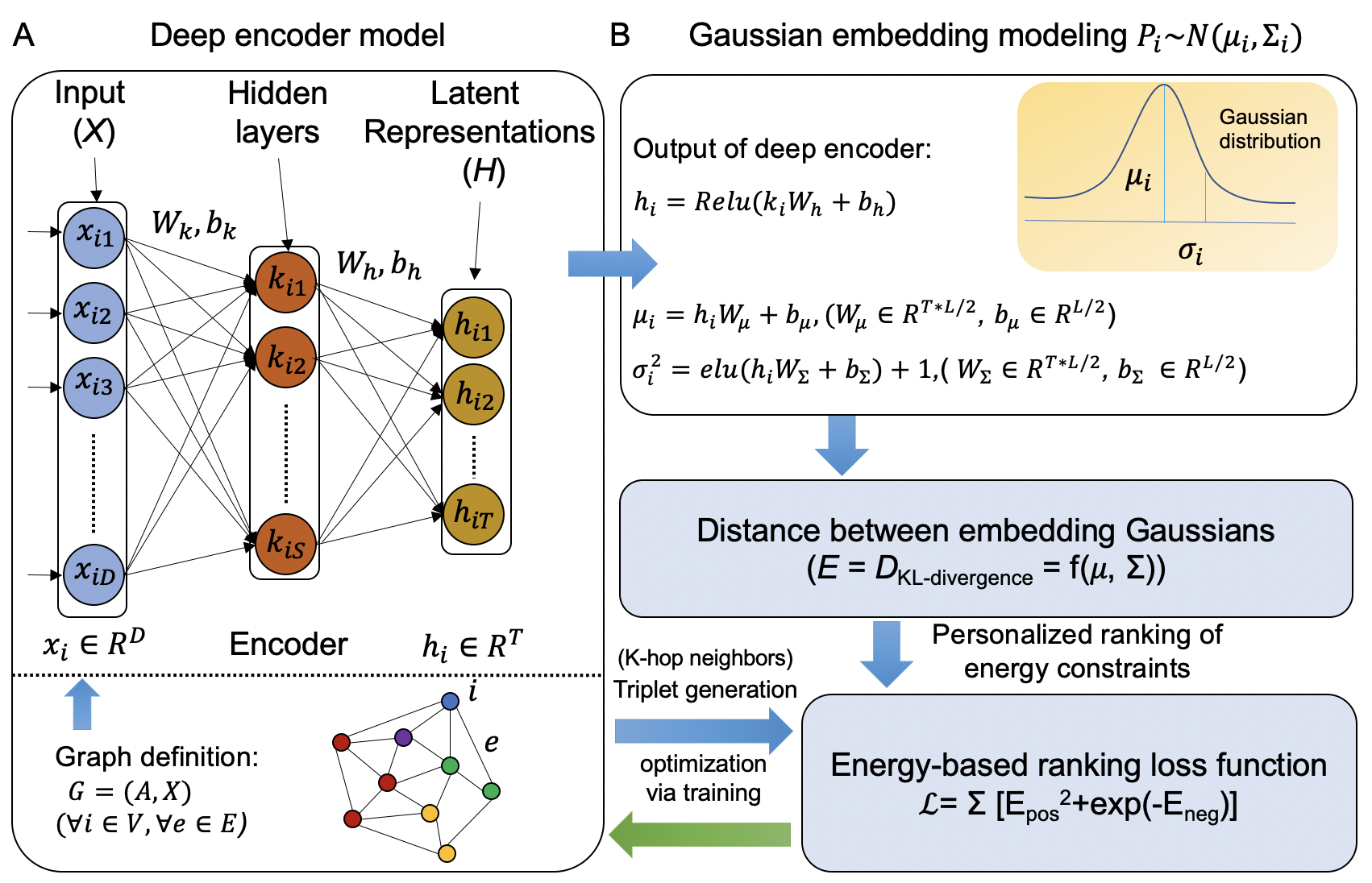


**S1 Fig. Illustration of the main workflow for the Graph2Gauss embedding approach**. (**A**) The node attribute matrix (*X*) is input to a deep encoder with a sequence of hidden layers (Graph2Gauss adopted single hidden layer), yielding the latent representations (*H*). Moreover, the node context (i.e., different hops) for each node can be identified based on the adjacency matrix (*A*) through computing k-hop neighbors ($k\geq2$) (see Eq.(2) below), e.g., $N_{ik}$ denotes the $k$-hop neighbors of node $i$. Sampling different hops for every node can benefit the preservation of multi-scale graph structure property (i.e., first-order proximity and second-order proximity) for graph embedding. Subsequently, the corresponding node triplet set ($D_{t}$) (see Eq. (3) below) for each node can be built by sampling different positive and negative node pairs based on the obtained node context (hops). *D_t_* is subsequently applied in conjunction with the attribute embedding output for learning the projection and optimize the Graph2Gauss model. **(B)** The output of the deep encoder is then fed into two independent feed-forward neural networks to obtain the mean ($\mu_{i}$) and diagonal variance ($\Sigma_{i}$) for a joint Gaussian distribution. Finally, the Graph2Gauss model is trained using an *energy-based triplet ranking loss* (Eq. (6)) that penalizes the ranking constraints presented in Eq (4). The dissimilarity (or energy) between Gaussian embeddings of different positive and negative node pairs sampled using *D_t_* can be computed using the *KL-divergence* (Eq. (5)).

Below, we provide details for some of the key components of the Gaussian embedding learning process.

*1) Node triplet generation*: Given a node *i*, its *k*-hop neighbors can be represented as $N_{ik}$in Eq (2), where *sp*(*i, j*) denotes the shortest path between node *i* and node *j* (if *i* and *j* are not reachable, it returns $\infty$); *K* is the maximum considered distance, usually $K > 2$ enables capturing high-order proximity. A triplet sample usually consists of anchor, positive, and negative nodes [1]; a set of valid triplets can be represented as in Eq ([3](#_2et92p0)), with $j_{k}\in N_{ik}$, $j_{l}\in N_{il}$ and *k < l*. Thus, for the triplet (*i,*$j_{k}$*,* $j_{l}$), node *i* is more similar to node $j_{k}$ than node $j_{l}$, and the node pair (*i,*$j_{k}$) denotes one positive pair, while the node pair (*i,*$j_{l}$) denotes one negative pair, which is generated for the energy-based ranking loss construction. Moreover, a “node-anchored sampling" strategy [2] provides an effective way for triplet generation that can help reduce the computational complexity in a large graph.

$N_{ik}=\left\{ j\in V \right|i \neq j,\min\left( sp\left( i,j \right), K \right)=k\}$ (2)

$D_{t}=\left\{ \left( i,j_{k},j_{l} \right) | sp\left( i,j_{k} \right)<sp\left( i,j_{l} \right) \right\}$ *.* (3)

*2) Network structure preservation via personalized ranking*: In order to capture the network structure properties at a multiscale level for graph embedding, personalized ranking of energy (similarity) constraints in Eq (4) are imposed to the latent node embeddings, *i.e.,* the respective energy (or distance) between embeddings of node *i* and each node in its *k*-hop neighbors (“positive energy”) is lower than the one between embeddings of node *i* and its (*k*+1)-hop neighbors (“negative energy”). Here, $E(P_{i}, P_{j})$ denotes the energy function between learned Gaussian distributions $(P_{i}, P_{j})$ for nodes *i* and *j*. The specifications of energy function can be seen below:

$E\left( P_{i},P_{k_{1}} \right)<E\left( P_{i},P_{k_{2}} \right)<\ldots<E\left( P_{i},P_{k_{K}} \right) \forall k_{1}\in N_{i1}, \forall k_{2}\in N_{i2},\ldots., \forall k_{K}\in N_{iK}$. (4)

*3) Similarity quantification for embedding Gaussians*: The energy function (*E*) in Eq ([4](#_tyjcwt)) is used to measure the distance between two nodes’ embedding Gaussian distributions ($P_{i}$*,* $P_{j}$) and score the triplets. Here (*i, j*) can be either positive node pairs or negative node pairs. Currently, most commonly used similarity metrics include the symmetric expected likelihood (*EL*), the Jensen-Shannon divergence (*JS*), the asymmetric KL-divergence (*KL*), and the *p*-th Wasserstein distance (*W_p_*). Different from the first two metrics, *KL* and *W_p_* can also handle directed graph embeddings while at the same time preserving the transitivity of nodes. The asymmetric “KL-divergence” energy is shown in Eq ([5](#_3dy6vkm)), where tr(.) denotes the trace of a matrix, and det(.) denotes the determinant. Smaller *E* represents that the nodes’ embedding Gaussians are more similar or closer to each other.


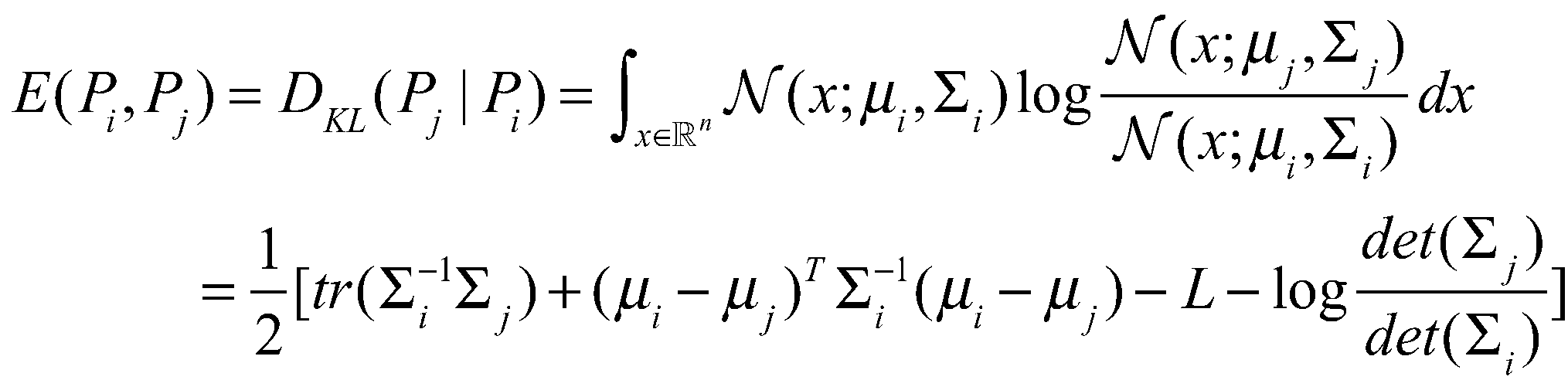
 (5)

*4) Energy-based ranking loss function*: Based on the aforementioned constraints on the respective energy between latent embeddings of adjacent *k*-hop neighbors of each node (e.g*.*, 2-hop vs. 3-hop of node *i*), in order to learn a graph embedding that satisfies the constraints, we adopt an energy-based learning approach [3]. That is, we build an energy-based ranking loss ($L$) over the sampled triplets ($D_{t}$) for penalizing the ranking errors, such that positive energy ($E_{ij_{k}}$) terms are always lower than negative energy ($E_{ij_{l}}$). $L$ usually consists of two parts: positive node pair energy ${(E}_{ij_{k}})$ and negative node pair energy ${(E}_{ij_{l}})$. “Margin-based ranking loss” is a frequently used loss function for graph embedding learning, however, the margin has to be manually selected before training. Thus, the “square-exponential loss” [12] representing negative pair energy as an exponential term has better performance in penalizing the ranking error automatically; the specific formula is given in Eq (6).


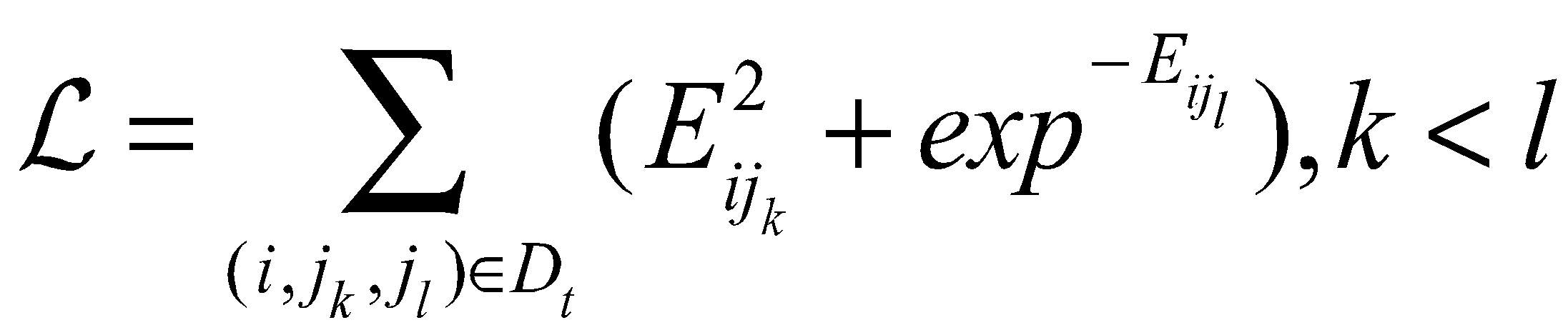
 (6)
