# Supplementary material for: A new Graph Gaussian embedding method for analyzing the effects of cognitive training
Source: PLoS Comput Biol. 2020 Sep 17;16(9):e1008186. doi: 10.1371/journal.pcbi.1008186 (PMC7524000; doi:10.1371/journal.pcbi.1008186)
Supplement: S2 Appendix — (DOCX) [file pcbi.1008186.s002.docx]

# S2 Appendix. Example of uncertainty quantification

To illustrate how Graph2Gauss, and specifically our proposed MG2G method, estimates uncertainty, we plot Gaussian distributions for several nodes (ROIs) of one patient in S2 Fig. The difference of two Gaussians is also a Gaussian with mean the difference of the two means and variance the sum of the two variances.


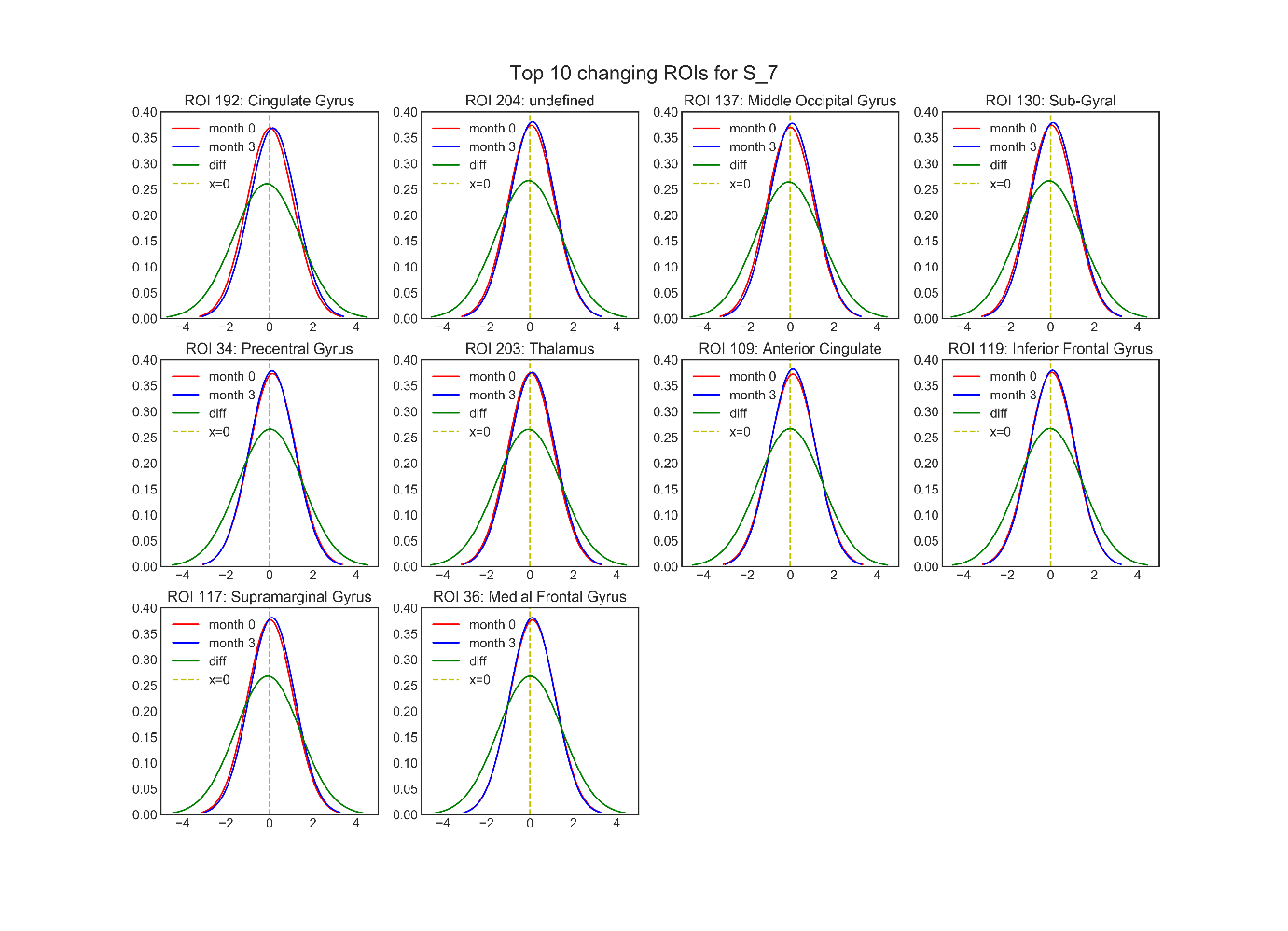


**S2 Fig. Gaussian distributions obtained from MG2G for one patient.** ROI-specific embedding Gaussian distributions before (red curve), after (blue curve) MDCT intervention, and the distribution of the difference (green curve). We only include the top-10 ROIs with the highest W2-distance.
