# Supplementary material for: A new Graph Gaussian embedding method for analyzing the effects of cognitive training
Source: PLoS Comput Biol. 2020 Sep 17;16(9):e1008186. doi: 10.1371/journal.pcbi.1008186 (PMC7524000; doi:10.1371/journal.pcbi.1008186)
Supplement: S3 Appendix — (DOCX) [file pcbi.1008186.s003.docx]

# S3 Appendix. Comparison of MG2G versus node2vec

We evaluated the performance of our proposed MG2G method against node2vec. S3 Fig. shows system-level changes for each patient using MG2G, and S4 Fig. gives the corresponding results using node2vec. The output of node2vec is deterministic and hence we can only compare mean values with MG2G.


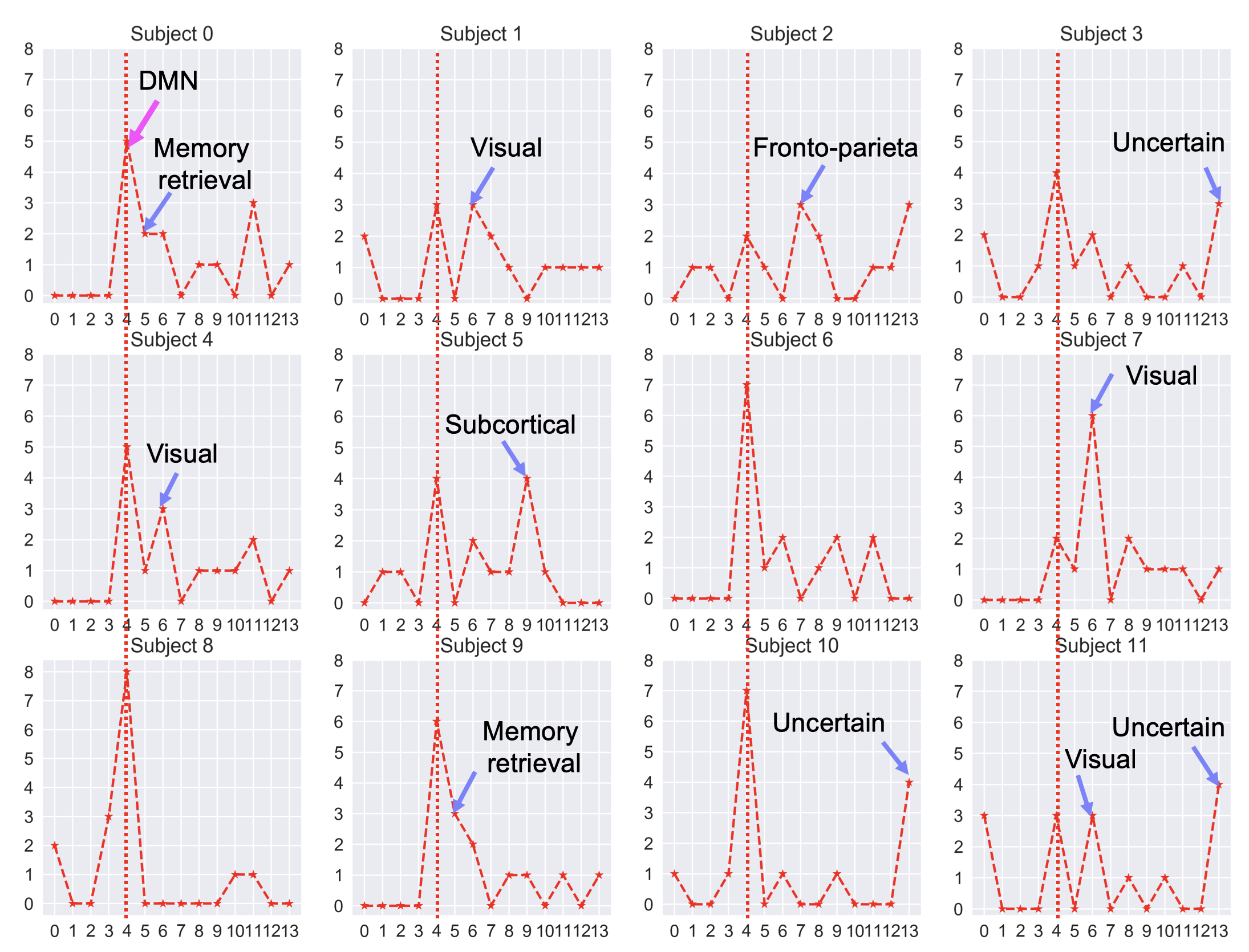


**S3 Fig. Quantification of system-level changes for all 12 patients before and after MDCT intervention based on MG2G**. The plots show the number of top-15 ROIs with the highest network alterations contained within different functional brain systems. System names same as in main text Fig 7.

**
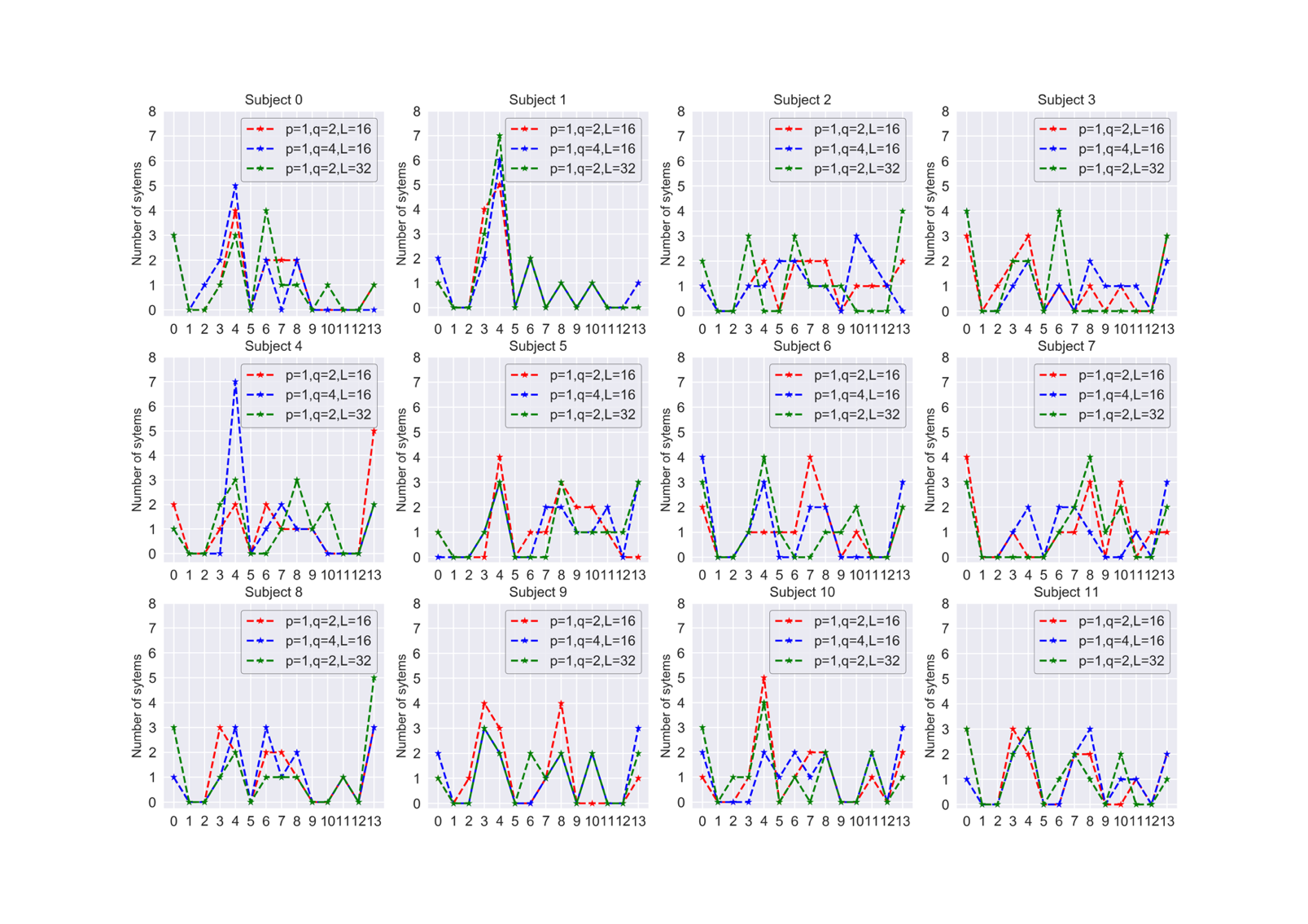
**

**S4 Fig. Quantification of system-level changes for all 12 patients before and after MDCT intervention based on different parameters of node2vec.** We tested performance with different embedding sizes (*L* = 16, 32) and p, q values in node2vec; these values control the neighborhood exploration in node2vec.
