# Supplementary material for: A new Graph Gaussian embedding method for analyzing the effects of cognitive training
Source: PLoS Comput Biol. 2020 Sep 17;16(9):e1008186. doi: 10.1371/journal.pcbi.1008186 (PMC7524000; doi:10.1371/journal.pcbi.1008186)
Supplement: S4 Appendix — (DOCX) [file pcbi.1008186.s004.docx]

# S4 Appendix. Example of reorganization index (RI) estimation

We estimated the RI for the different ROIs comprising the neural system ‘sensory/somatomotor hand’ (SSH). Both ROIs (8 and 28) that achieved the highest RI values are located in the precentral gyrus of the right hemisphere. The majority of the ROIs had positive RI values, indicating extensive reorganization of the SSH neural system. More details are shown in S5 Fig.


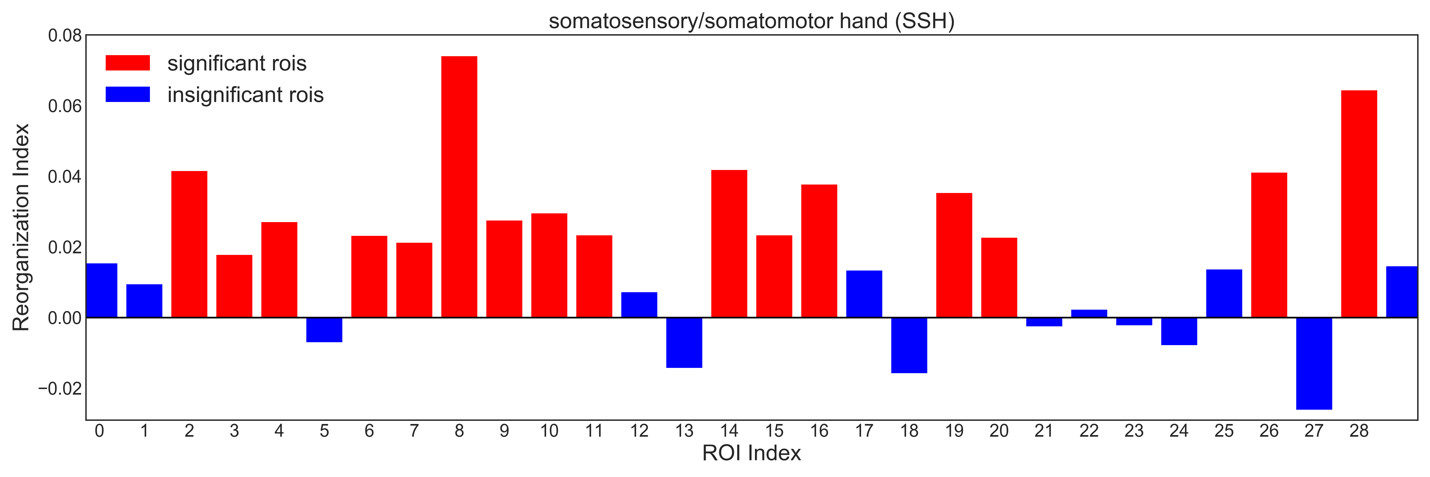


**S5 Fig. Reorganization index results for 30 ROIs in the ‘*somatosensory/somatomotor hand’* functional brain system**. A large number of ROIs had significant RI (red bars; p<0.05, FDR corrected). System name abbreviations same as in S1 Table.
