# Supplementary material for: A new Graph Gaussian embedding method for analyzing the effects of cognitive training
Source: PLoS Comput Biol. 2020 Sep 17;16(9):e1008186. doi: 10.1371/journal.pcbi.1008186 (PMC7524000; doi:10.1371/journal.pcbi.1008186)
Supplement: S5 Appendix — (DOCX) [file pcbi.1008186.s005.docx]

# S5 Appendix. Interpretation of the reorganization index (RI) in the embedding latent space

As we mentioned in the main text, a positive RI value for a given brain region suggests network alterations due to the intervention. This is because the dots would tend to cluster in the same region in the embedded space for within-intervention subjects (both no intervention, or both intervention), but would map to distant areas for between-intervention subjects.


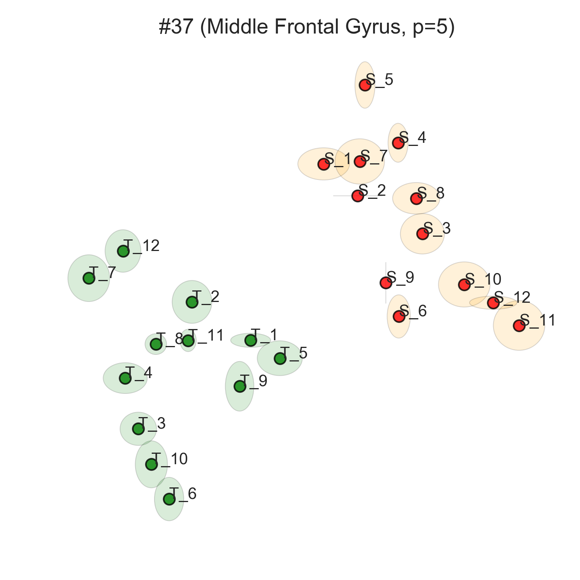

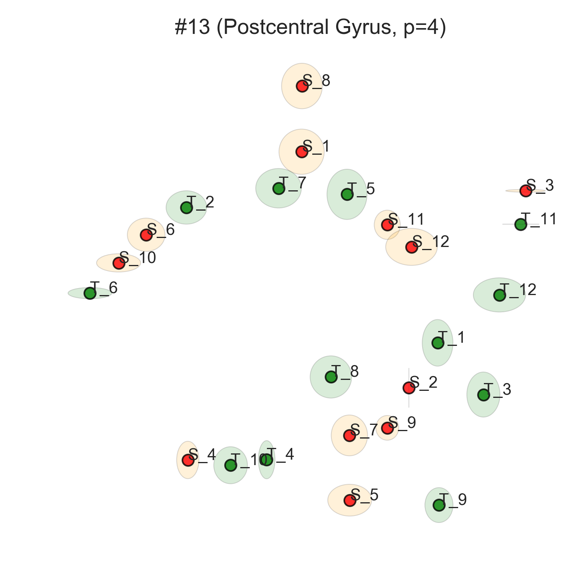


**S6 Fig. 2D visualization for different brain regions embeddings before and after intervention.** From left to right are plots for significant changing ROI (# 37, Middle Frontal Gyrus) with RI>0 and insignificant changing ROI (# 13, postcentral gyrus) with RI<0. The dots represent the Gaussian embedding mean values of two specific ROI for 12 different subjects before (yellow color) and after intervention (green color), while the ellipses represent the uncertainty (variance) information of different ROIs.
