# Supplementary material for: A new Graph Gaussian embedding method for analyzing the effects of cognitive training
Source: PLoS Comput Biol. 2020 Sep 17;16(9):e1008186. doi: 10.1371/journal.pcbi.1008186 (PMC7524000; doi:10.1371/journal.pcbi.1008186)
Supplement: S6 Appendix — (DOCX) [file pcbi.1008186.s006.docx]

# S6 Appendix. Comparison with and without global signal regression

We have applied the MG2G model to learn functional brain network embeddings based on fMRI time-series extracted with and without global signal regression, and evaluated the ROI-wise W2 distances for two different randomly selected subjects. The corresponding comparison results are shown in S6 Fig. below. In the two plots below, the orange curves represent the W2 distance results without global signal regression, while the blue curves show the results with global signal regression.


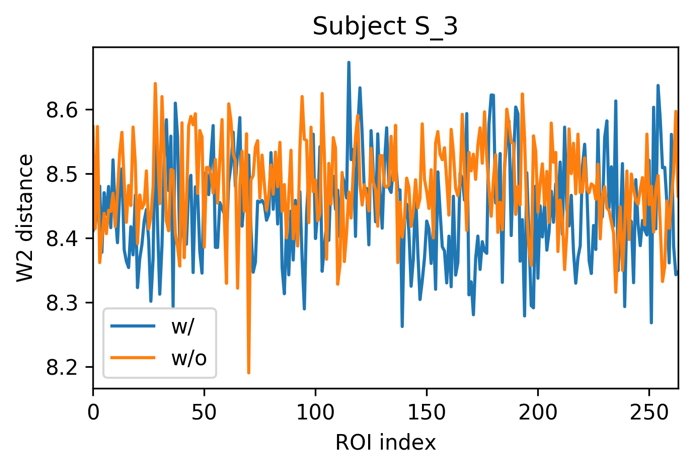

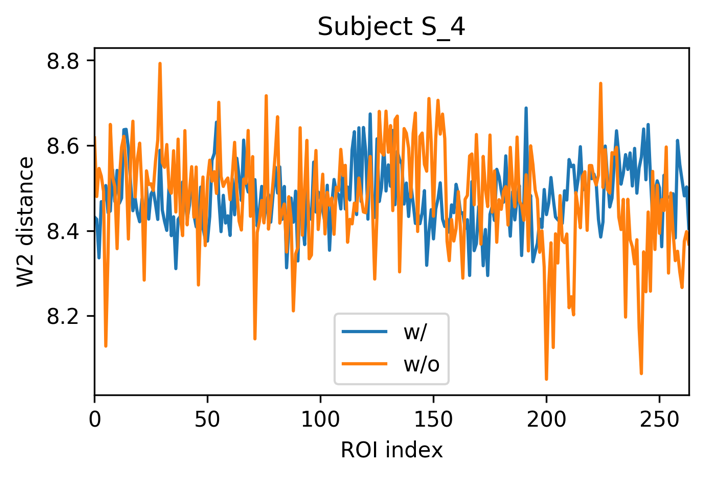


**S7 Fig. W2 distance versus ROI for two randomly selected subjects based on fMRI brain networks, computed from time-series with (w/) and without (w/o) global signal regression.**

Additionally, the top 15 changing brain regions for subject 3 and subject 4 can be found in S1 Table. Based on the consistent ROIs captured by using ROI-wise W2 distance, these results demonstrate that overall our model is robust in detecting the most significantly changing regions after MDCT training, irrespective of the global signal regression. An exception, however, is seen in subject 4’s results in S1 Table.; using time-series without global signal regression failed to detect the “Parahippocampa Gyrus” regions. This might be due to some hidden noise in the raw data.

**S1 Table. Top 15 changing ROIs based on Gaussian embeddings learned from signals with and without global signal regression operation** (The consistent ROIs are marked in red color while black color denotes brain regions with inconsistent results in the two top changing ROI lists obtained from using global signal regression and without using global signal regression in the extracted signals)

| Patient ID | w/ global signal regression | w/o global signal regression |
| --- | --- | --- |
| Subject 3 | 'Middle Frontal Gyrus'  'Declive'  'Precentral Gyrus'  'Superior Frontal Gyrus'  'Cingulate Gyrus'  'Inferior Parietal Lobule'  'Inferior Occipital Gyrus'  'Anterior Cingulate'  'Middle Frontal Gyrus'  'Declive'  'Medial Frontal Gyrus'  'Middle Frontal Gyrus'  'Cingulate Gyrus'  'Superior Frontal Gyrus'  'Middle Temporal Gyrus' | 'Middle Temporal Gyrus'  'Precentral Gyrus'  'Superior Frontal Gyrus'  'Inferior Parietal Gyrus'  'Middle Frontal Gyrus'  'Superior Temporal Gyrus'  'Middle Frontal Gyrus'  'Fusiform Gyrus'  'Extra-Nuclear'  'Insula'  'Insula'  'Medial Frontal Gyrus'  'undefined'  'Middle Temporal Gyrus'  'Cingulate Gyrus' |
| Subject 4 | Uncus  Superior Frontal Gyrus  Parahippocampa Gyrus  Middle Temporal Gyrus  Middle Frontal Gyrus  Lingual Gyrus  Postcentral Gyrus  Cuneus  Parahippocampa Gyrus  Middle Temporal Gyrus  Inferior Frontal Gyrus  Fusiform Gyrus  Superior Temporal Gyrus  Precuneus  Cingulate Gyrus | Postcentral Gyrus  Postcentral Gyrus  Lingual Gyrus  Posterior Cingulate  Cuneus  Middle Frontal Gyrus  Lingual Gyrus  Middle Temporal Gyrus  Sub-Gyral  Insula  Cuneus  Precuneus  Posterior Cingulate  Middle Temporal Gyrus  Postcentral Gyrus |

w/: with global signal regression; w/o: without global signal regression.
