# Supplementary figures and images for: A new Graph Gaussian embedding method for analyzing the effects of cognitive training
Source: PLoS Comput Biol. 2020 Sep 17;16(9):e1008186. doi: 10.1371/journal.pcbi.1008186 (PMC7524000; doi:10.1371/journal.pcbi.1008186)

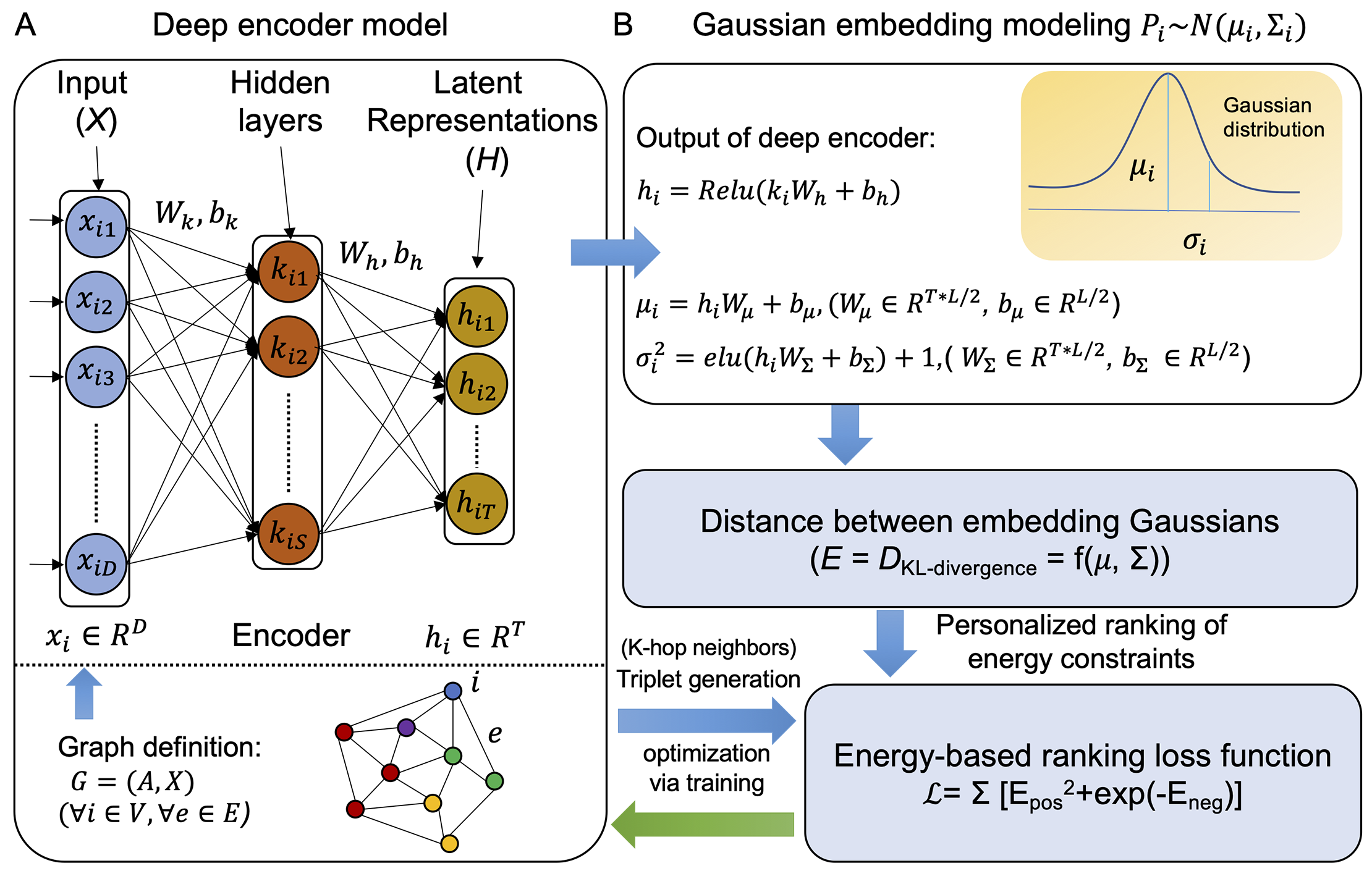

Supplement: S1 Fig — (TIF) [file pcbi.1008186.s008.tif]

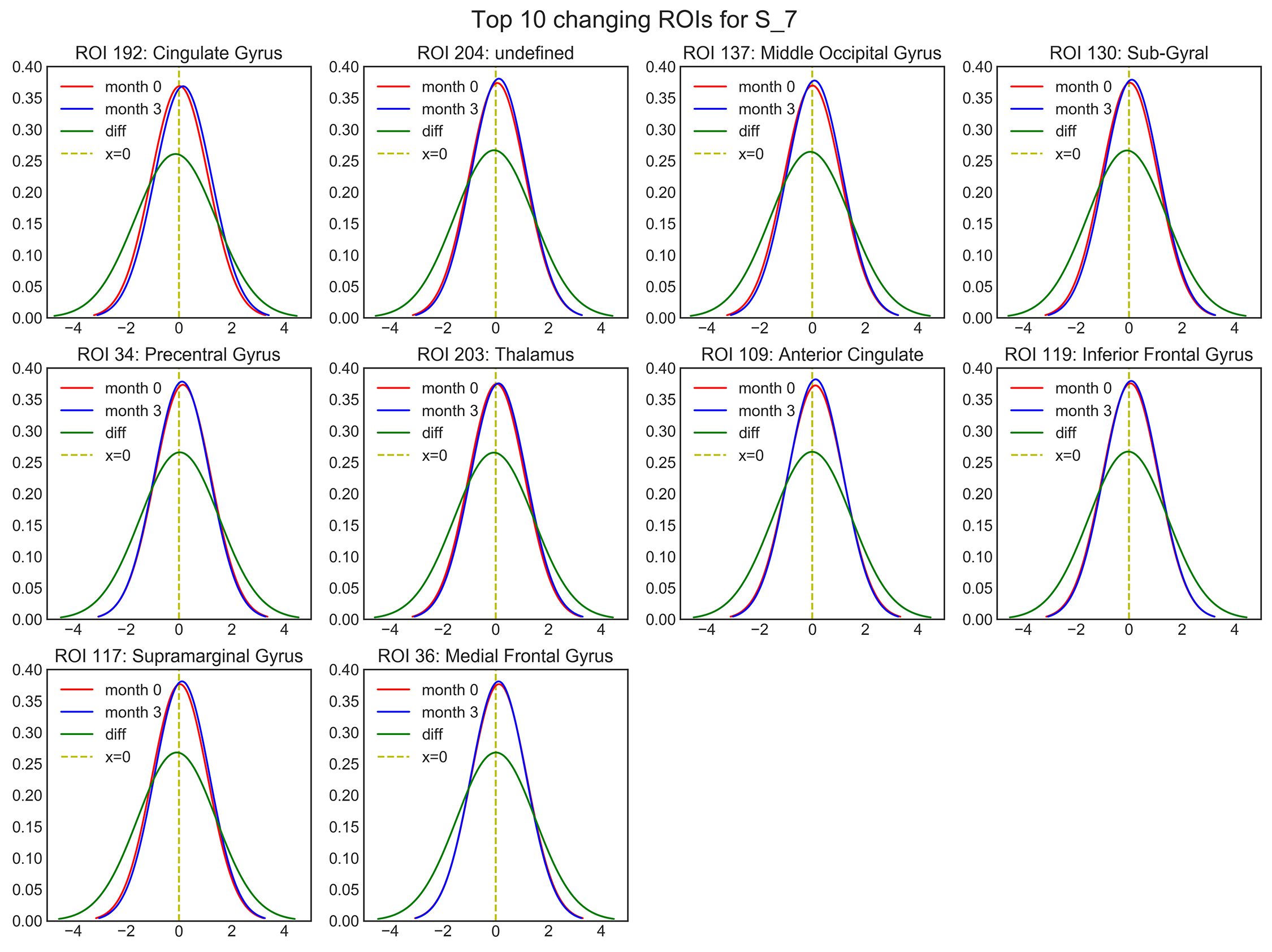

Supplement: S2 Fig — (TIF) [file pcbi.1008186.s009.tif]

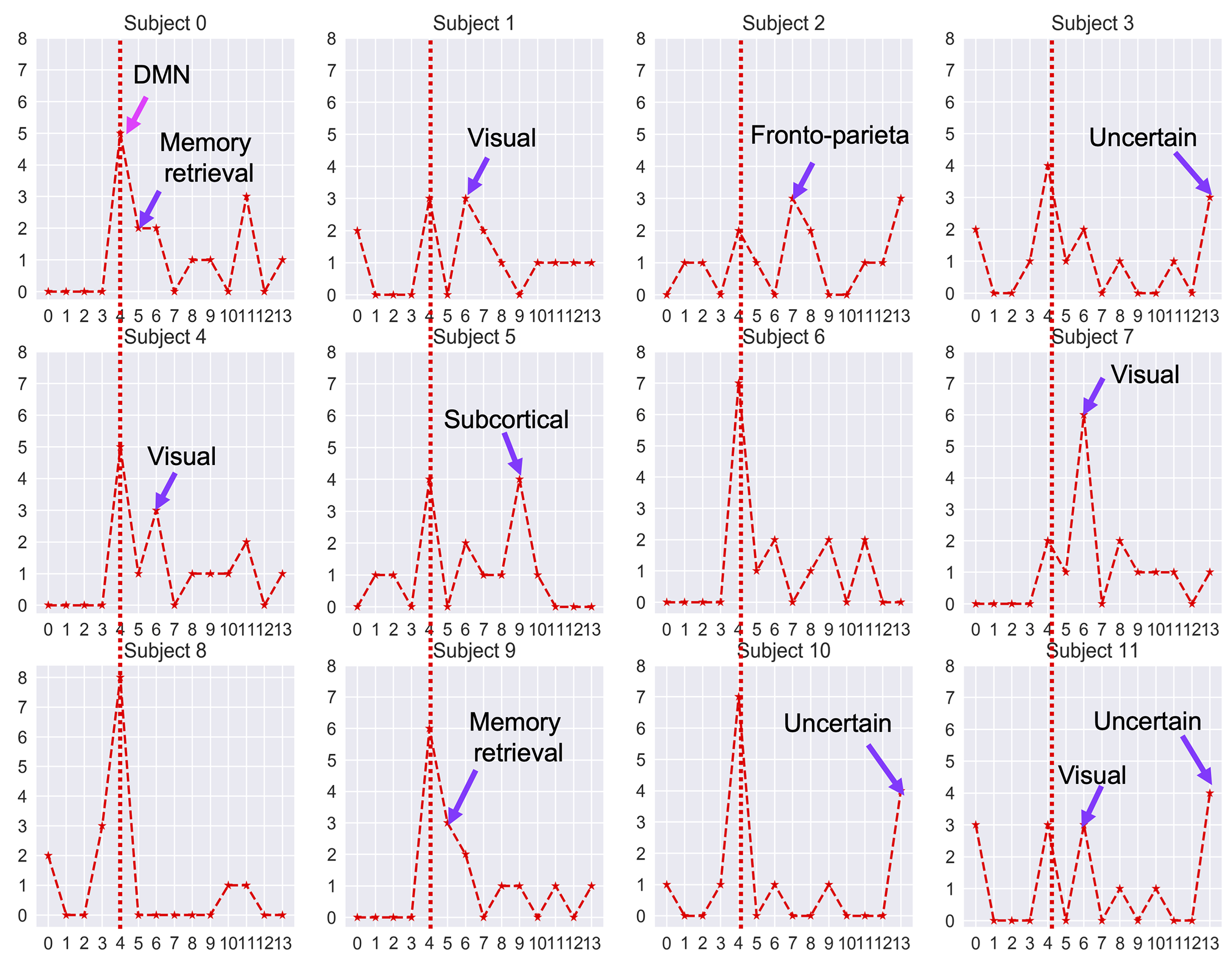

Supplement: S3 Fig — (TIF) [file pcbi.1008186.s010.tif]

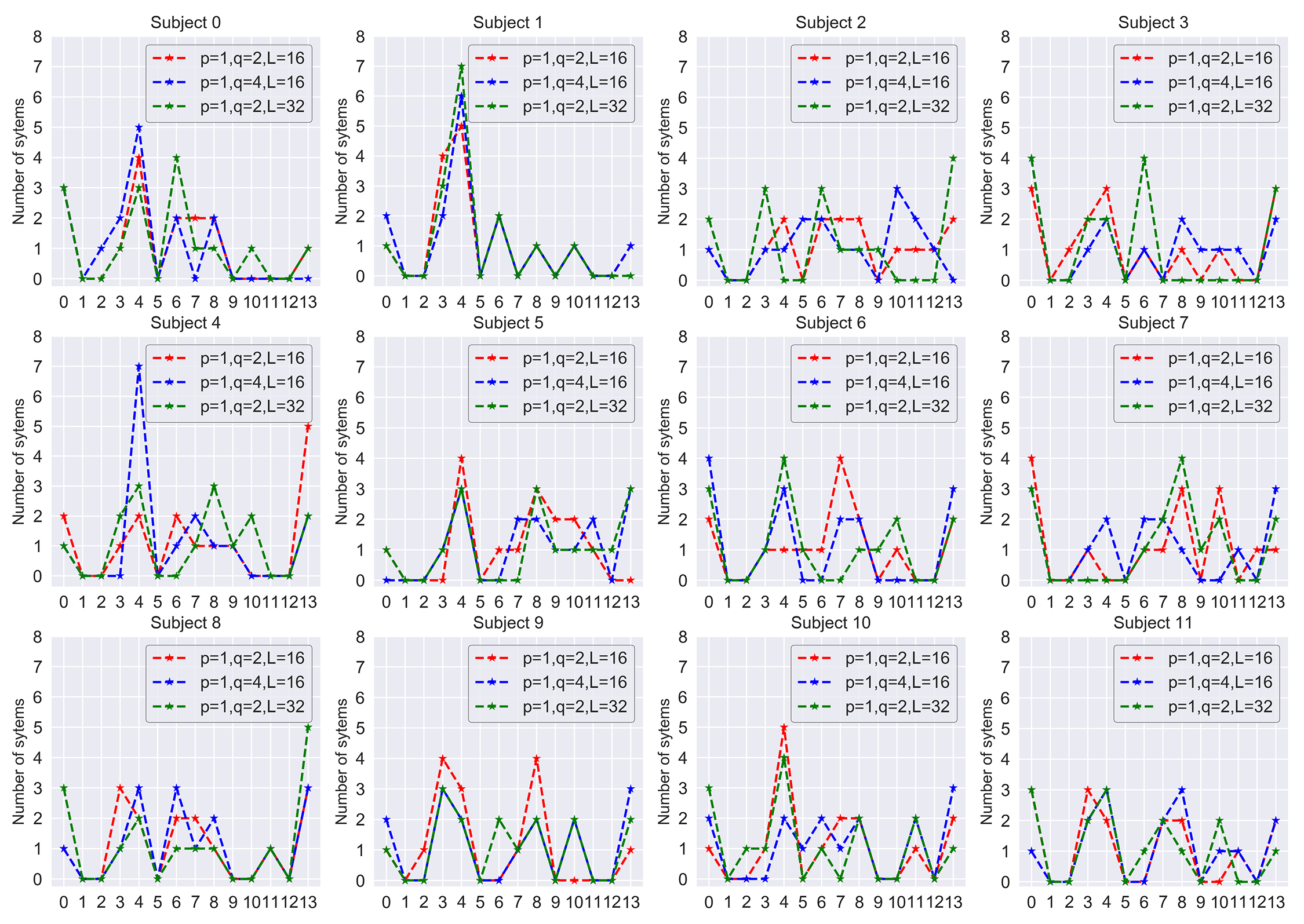

Supplement: S4 Fig — (TIF) [file pcbi.1008186.s011.tif]

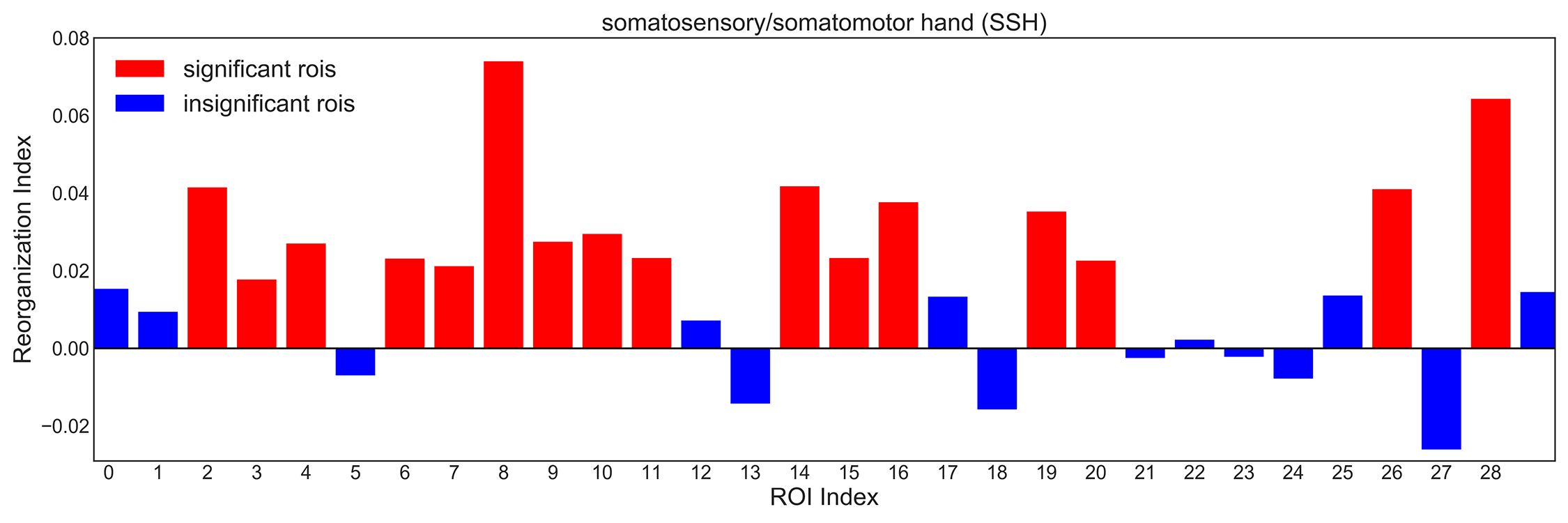

Supplement: S5 Fig — (TIF) [file pcbi.1008186.s012.tif]

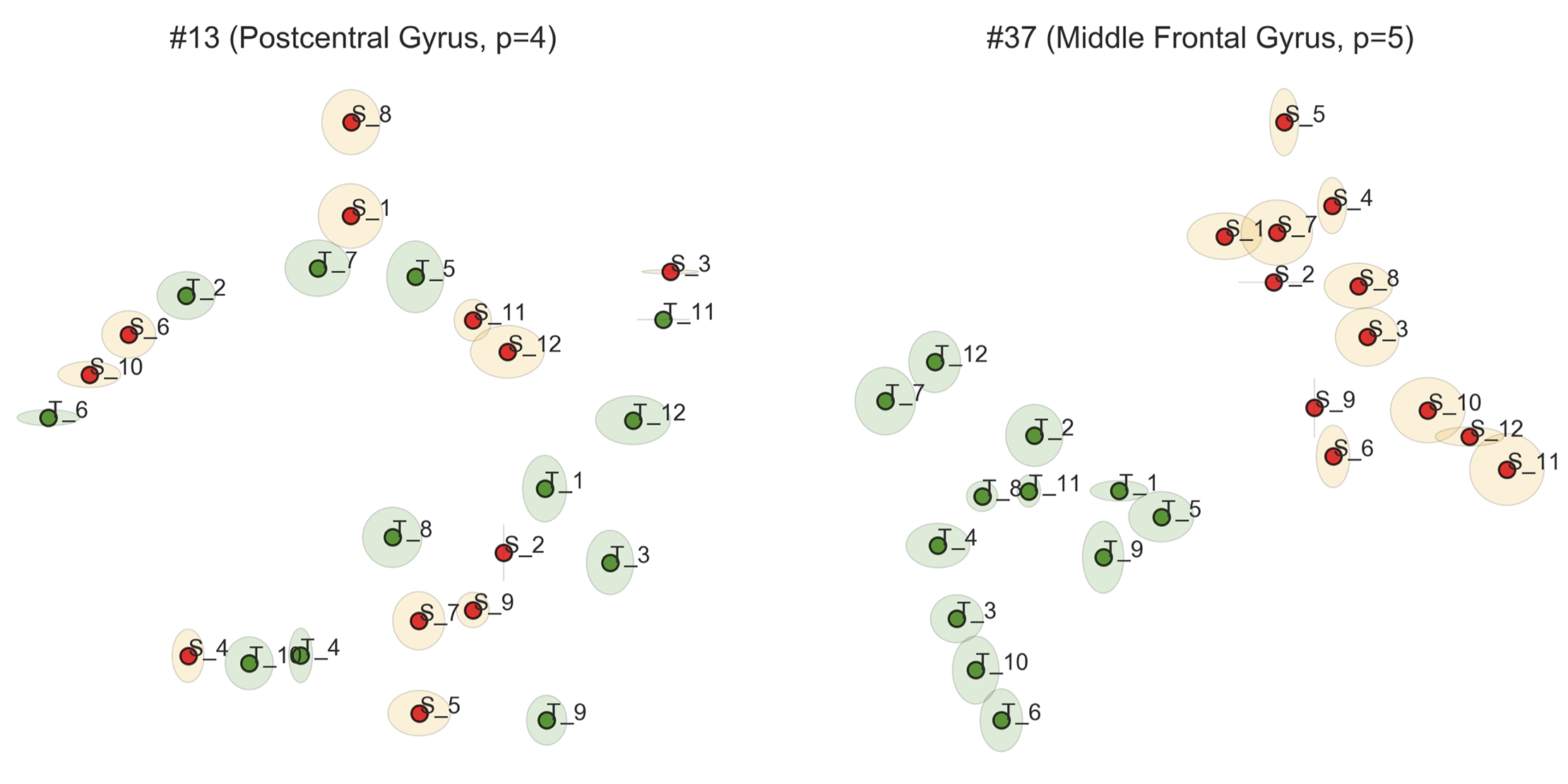

Supplement: S6 Fig — (TIF) [file pcbi.1008186.s013.tif]

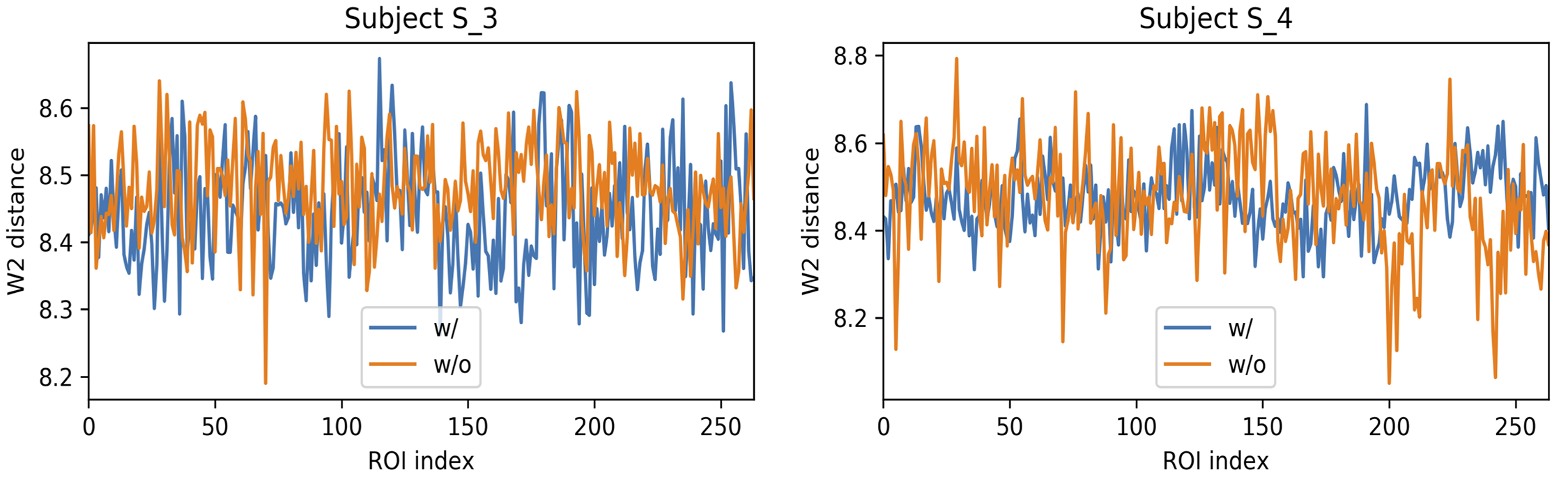

Supplement: S7 Fig — (TIF) [file pcbi.1008186.s014.tif]
