# Supplementary material for: A new Graph Gaussian embedding method for analyzing the effects of cognitive training
Source: PLoS Comput Biol. 2020 Sep 17;16(9):e1008186. doi: 10.1371/journal.pcbi.1008186 (PMC7524000; doi:10.1371/journal.pcbi.1008186)
Supplement: S1 Table — (DOCX) [file pcbi.1008186.s015.docx]

**S1 Table. Top 15 changing ROIs based on Gaussian embeddings learned from signals with and without global signal regression operation** (The consistent ROIs are marked in red color while black color denotes brain regions with inconsistent results in the two top changing ROI lists obtained from using global signal regression and without using global signal regression in the extracted signals)

| Patient ID | w/ global signal regression | w/o global signal regression |
| --- | --- | --- |
| Subject 3 | 'Middle Frontal Gyrus'  'Declive'  'Precentral Gyrus'  'Superior Frontal Gyrus'  'Cingulate Gyrus'  'Inferior Parietal Lobule'  'Inferior Occipital Gyrus'  'Anterior Cingulate'  'Middle Frontal Gyrus'  'Declive'  'Medial Frontal Gyrus'  'Middle Frontal Gyrus'  'Cingulate Gyrus'  'Superior Frontal Gyrus'  'Middle Temporal Gyrus' | 'Middle Temporal Gyrus'  'Precentral Gyrus'  'Superior Frontal Gyrus'  'Inferior Parietal Gyrus'  'Middle Frontal Gyrus'  'Superior Temporal Gyrus'  'Middle Frontal Gyrus'  'Fusiform Gyrus'  'Extra-Nuclear'  'Insula'  'Insula'  'Medial Frontal Gyrus'  'undefined'  'Middle Temporal Gyrus'  'Cingulate Gyrus' |
| Subject 4 | Uncus  Superior Frontal Gyrus  Parahippocampa Gyrus  Middle Temporal Gyrus  Middle Frontal Gyrus  Lingual Gyrus  Postcentral Gyrus  Cuneus  Parahippocampa Gyrus  Middle Temporal Gyrus  Inferior Frontal Gyrus  Fusiform Gyrus  Superior Temporal Gyrus  Precuneus  Cingulate Gyrus | Postcentral Gyrus  Postcentral Gyrus  Lingual Gyrus  Posterior Cingulate  Cuneus  Middle Frontal Gyrus  Lingual Gyrus  Middle Temporal Gyrus  Sub-Gyral  Insula  Cuneus  Precuneus  Posterior Cingulate  Middle Temporal Gyrus  Postcentral Gyrus |

w/: with global signal regression; w/o: without global signal regression.
