# Supplementary material for: A new Graph Gaussian embedding method for analyzing the effects of cognitive training
Source: PLoS Comput Biol. 2020 Sep 17;16(9):e1008186. doi: 10.1371/journal.pcbi.1008186 (PMC7524000; doi:10.1371/journal.pcbi.1008186)
Supplement: S2 Table — (DOCX) [file pcbi.1008186.s016.docx]

**S2 Table. Functional brain atlas information.**

| NO. | ROI range | System | Abbreviation |
| --- | --- | --- | --- |
| 1 | 0-29 | Sensory/somatomotor Hand | SSH |
| 2 | 30-34 | Sensory/somatomotor Mouth | SSM |
| 3 | 35-48 | Cingulo-opercular Task Control | CoTC |
| 4 | 49-61 | Auditory | Audit |
| 5 | 62-119 | Default mode | DMN |
| 6 | 120-124 | Memory retrieval | MemRt |
| 7 | 125-155 | Visual | Vis |
| 8 | 156-180 | Fronto-parietal Task Control | FpTC |
| 9 | 181-198 | Salience | Sal |
| 10 | 199-211 | Subcortical | SubCt |
| 11 | 212-220 | Ventral attention | VenAtt |
| 12 | 221-231 | Dorsal attention | DorsAtt |
| 13 | 232-235 | Cerebellar | Cerebl |
| 14 | 236-263 | Uncertain | Uncert |
